# Plasma metabolites, dietary intakes, and breast cancer incidence: a prospective case-cohort study in NutriNet-Santé

**DOI:** 10.1016/j.ajcnut.2026.101342

**Published:** 2026-05-23

**Authors:** Ashley H Larnder, Rachel A Murphy, Bernard Srour, Nicolas Déchamp, Pilar Galan, Serge Hercberg, Laurent Bourhis, Caridad Díaz, Olga Genilloud, Mathilde Touvier, Mélanie Deschasaux-Tanguy

**Affiliations:** 1 School of Population and Public Health, University of British Columbia, Vancouver, BC, Canada; 2 Population Health Sciences, BC Cancer Research Institute, Vancouver, BC, Canada; 3 Université Sorbonne Paris Nord and Université Paris Cité, INSERM, INRAE, CNAM, Centre of Research in Epidemiology and StatisticS (CRESS), Nutritional Epidemiology Research Team (EREN), Bobigny, France; 4 Nutrition And Cancer Research (NACRe) Network, Jouy-en-Josas, France; 5 Centro de Excelencia en Investigación de Medicamentos Innovadores en Andalucía, Armilla, Granada, Spain

**Keywords:** breast cancer incidence, case-cohort, metabolomics, diet, nutrition, mediation analysis

## Abstract

**Background:**

Breast cancer (BC) is the most common cancer in females. Combining metabolomic and dietary data provides an opportunity to explore metabolic pathways underlying BC risk, but few prospective studies have examined these relationships.

**Objectives:**

This study aimed to identify plasma metabolites associated with incident BC, explore diet–metabolite associations, and assess whether metabolites mediate diet–BC associations.

**Methods:**

Fifty-three plasma metabolites were measured by liquid chromatography–mass spectrometry in females from a prospective case-cohort nested within the French NutriNet-Santé study (2762 subcohort members, 306 total BC cases; median age 53.6 y). Dietary intake was assessed using repeated 24-h dietary records. Principal component (PC) analysis derived metabolite and dietary patterns. Cox models estimated hazard ratios (HRs) for metabolite–BC associations, adjusted for established risk factors. ElasticNet and linear regression identified dietary predictors of metabolites. Mediation analysis evaluated whether metabolites mediated diet–BC associations.

**Results:**

A metabolite pattern enriched in conjugated bile acids (PC04) was positively associated with BC [HR: 1.14; 95% confidence interval (CI): 1.00, 1.30], including glycohyocholic acid (HR: 1.10; 95% CI: 1.03, 1.17), glycoursodeoxycholic acid (HR: 1.03; 95% CI: 1.00, 1.06), and taurocholic acid (HR: 1.04; 95% CI: 1.00, 1.09). A pattern enriched in unconjugated bile acids was inversely associated (HR: 0.86; 95% CI: 0.75, 0.99), as was linoleyl-carnitine (HR: 0.81; 95% CI: 0.68, 0.96) and, among premenopausal females, lysophosphatidylcholine (18:1) (HR: 0.60; 95% CI: 0.38, 0.96). Several dietary factors were associated with these metabolites; for example, vitamin K intake was inversely associated with glycohyocholic acid (β: ‒0.12 per standard deviation, 125.1 *μ*g/d) and PC04 (β: ‒0.07). Mediation analysis indicated vitamin K intake was inversely associated with BC (HR: 0.82; 95% CI: 0.68, 0.93), with reductions in glycohyocholic acid and PC04 each explaining ∼4% of this association.

**Conclusions:**

BC incidence was positively associated with conjugated bile acids, including the novel glycohyocholic acid, and inversely associated with unconjugated bile acids, suggesting a potential role for bile acid conjugation in carcinogenesis. Multiple dietary factors were linked to these metabolites, highlighting potential metabolic pathways connecting diet and BC.

This trial was registered at clinicaltrials.gov as NCT03335644.

## Introduction

Breast cancer is the most commonly diagnosed cancer among females worldwide [1,2]. Despite advances in early detection and treatment that have reduced mortality, the overall disease burden remains high. Established clinical (e.g., age, breast density, reproductive history) and environmental risk factors explain only part of breast cancer incidence, leaving a large proportion of risk unexplained [[3], [4], [5]]. This highlights the need to better understand disease etiology and underlying biological mechanisms.

Altered metabolism can promote breast cancer development and progression through mechanisms such as increased glycolysis fueling tumor cell proliferation, elevated fatty acid synthesis supporting hormone production, and shifts in amino acid and lipid pathways remodeling the breast tissue microenvironment [6,7]. Metabolomics offers a strategic tool to detect such alterations, as even modest shifts in enzyme activity or gene expression can lead to meaningful differences in metabolite concentrations, making this a promising approach for measuring biological changes related to cancer [7].

Several prospective studies have reported differences in pre-diagnosis plasma or serum metabolomic profiles between females who later develop incident breast cancer and those who remain cancer-free [[8], [9], [10], [11], [12], [13], [14]]. Reported metabolites include sex-steroid hormones [12], amino acids [14], catechols, and pyridine derivatives [13]; however, findings have been heterogeneous across studies. This inconsistently likely reflects methodological differences, including limited overlap in metabolite panels, variation in metabolomics platforms, and incomplete reporting of metabolites assessed in untargeted analyses, which complicates comparisons and replication across populations. In addition, most studies have focused on metabolite–disease associations in isolation, without clarifying how these metabolites relate to upstream exposures such as diet, limiting insights into underlying etiologic pathways.

Metabolomic profiles reflect both endogenous physiology and external exposure, including diet [15]. Although diet is hypothesized to influence breast cancer through metabolic and hormonal pathways—such as adiposity, insulin resistance, circulating hormones, and inflammation [15]—direct associations between diet and breast cancer have generally been modest; diet-related metabolic changes may attenuate or mask direct diet effects when metabolites are not accounted for. Nutritional metabolomics provides a framework to bridge this gap by identifying metabolites that both reflect dietary intake and are associated with disease risk. However, few prospective studies have jointly evaluated diet, metabolites, and breast cancer with an integrated analytical framework. Existing studies, including those focused on diet-related metabolites [15], have typically predefined metabolites based on dietary correlations and have not formally evaluated whether these metabolites mediate diet–breast cancer associations, nor have they accounted for the complex, multidimensional nature of dietary exposures with statistical modeling approaches.

To address these gaps, this study applies an integrative, metabolite-first approach within a prospective design. It first identifies plasma metabolites associated with incident breast cancer, then evaluates their dietary determinants (nutrients, foods) using multivariable methods that account for dietary complexity, and finally assesses whether these metabolites mediate diet–breast cancer associations. By embedding metabolite analyses within a framework linking diet, metabolism, and disease, this study aims to provide insight into potential metabolic pathways underlying breast cancer etiology.

## Methods

### Study design and data collection

This study included participants from the NutriNet-Santé study, a web-based prospective, longitudinal cohort (clinicaltrials.gov: NCT03335644). Launched in May 2009, the study continuously enrolls volunteers from the general French population aged >15 y through vast multimedia campaigns. All participants provide informed consent. This study was approved by the International Research Board of the French Institute for Health and Medical Research (international research board INSERM number 0000388FWA00005831) and the Comité National Informatique et Liberté (numbers 908450 and 909216).

Upon enrolment, participants were invited to complete self-administered questionnaires on health (e.g., personal and family history of disease, medication use), physical activity (international physical activity questionnaire [16,17]), lifestyle and sociodemographics (e.g., education level, smoking status and number of pack-years, number of children), and completed 24-h dietary records (described in more detail below). Every 6 mo, dietary records were collected, and participant health status was reassessed by a checkup questionnaire. At any time during follow-up, participants could also report a health event, a new treatment, or a medical examination via a dedicated and secured online platform. Additional details on the NutriNet-Santé cohort and data collection have been described previously [18].

Every reported cancer case was adjudicated by an expert medical committee, based on medical and anatomopathological reports collected from the participants, their physicians, and/or hospitals. Cases were classified using the International Classification of Diseases, 11th Revision[19]. The NutriNet-Santé study also had access to databases from the national health data system. Linkage of the cohort to the national health insurance medical database (Système National d’Information Inter-Régimes de l’Assurance Maladie) and the French cause-specific mortality registry (Centre d'épidémiologie sur les causes médicales de décès) enabled the completion of death and health data and limited potential bias from unreported cancer cases.

A subset of participants from the NutriNet-Santé cohort attended a clinical visit at a local collection center between 2011 and 2014, where trained personnel collected blood samples and anthropometric measurements (e.g., height, waist circumference). Blood was collected using lithium-heparin tubes with plasma separation gel plugs and stored at –80˚C in the NutriNet-Santé BioBank (University Sorbonne Paris Nord, Bobigny, France). Among 17,603 participants who provided fasting blood samples at a clinical visit and had ≥2 dietary records available prior to the clinical visit (the “original sample”), a case-cohort sample was established. This comprised a subcohort of 4200 participants randomly selected to be representative of the original sample, along with all incident cases of cancer, cardiovascular disease, type 2 diabetes, and mortality occurring among the remaining participants between the date of blood draw and 1 October, 2022 (“cases outside the subcohort”). The case-cohort design allows investigation of multiple outcomes while minimizing the costs associated with processing all the biological specimens [20].

**FIGURE 1:**
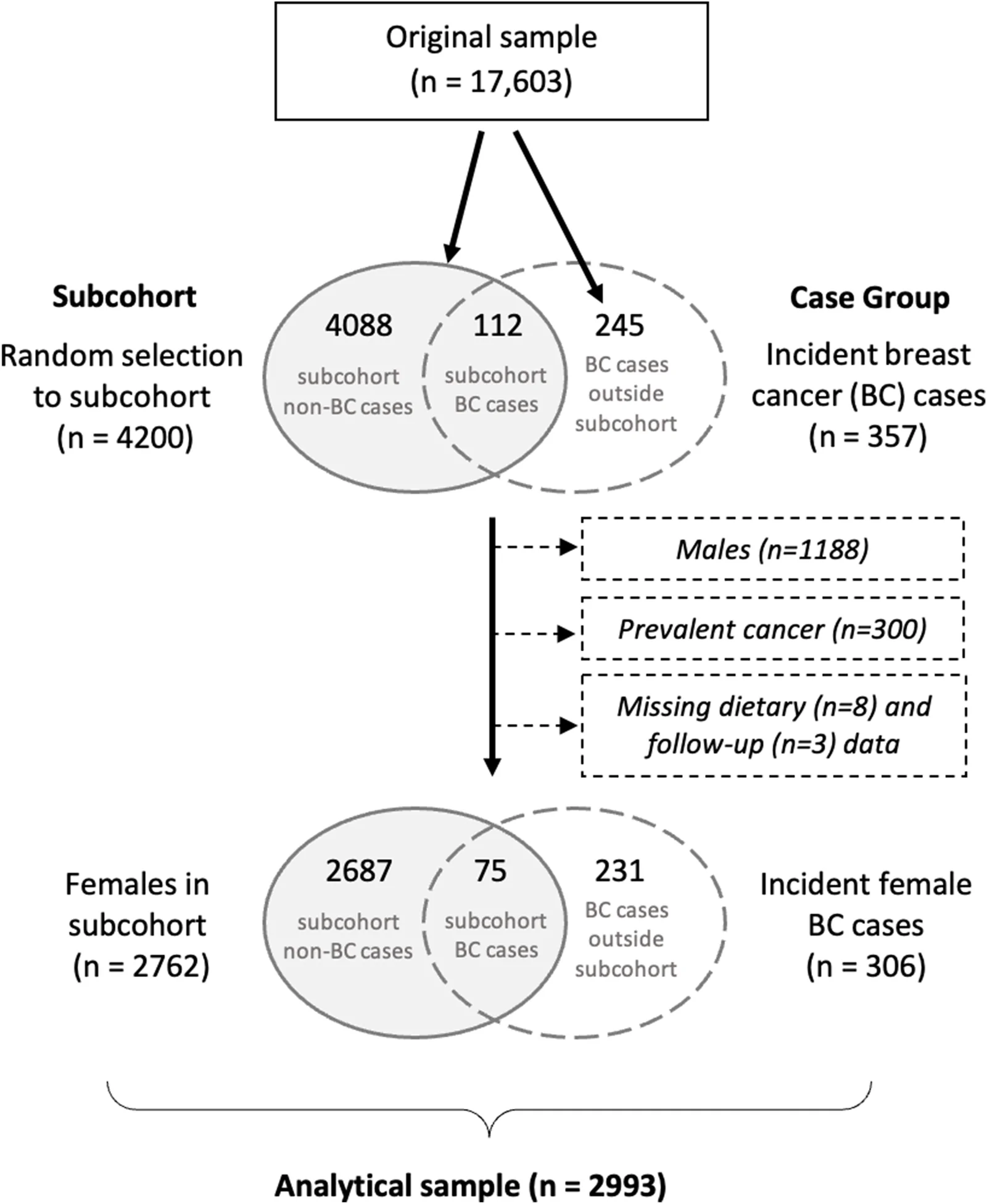
Flowchart of the case-cohort sample of females nested in the NutriNet-Santé prospective cohort study. Prevalence and incidence were considered relative to the date of clinical visit for each participant (baseline). Dotted lines indicate participants who were excluded. BC, breast cancer.

The present analysis focused on incident breast cancer. The analytical sample included participants from the subcohort and all additional breast cancer cases identified outside the subcohort. Participants were excluded if they were male, had a prevalent cancer at the clinical visit, or had a missing dietary or follow-up date. The final analytical sample included 2762 females in the subcohort and 306 incident breast cancer cases (75 from the subcohort, 231 from outside the subcohort). A detailed overview of the sample selection is shown in Figure 1. Participants contributed person-time from the date of clinical visit (baseline) until either the date of event (breast cancer diagnosis) or censor (other cancer diagnosis, death, lost to follow-up, or study end point: 1 October, 2022).

### Metabolomics

Plasma samples were collected at clinical appointments between 2011 and 2014 and analyzed in 13 batches using targeted metabolomics by Fundación MEDINA. A custom panel of 53 metabolites was selected to capture a broad range of compounds involved in metabolic processes linking dietary intake to health outcomes, with the full list provided in Supplementary Table 1.

Metabolite analysis was performed using liquid chromatography (Agilent 1290LC, Agilent Technologies) in reverse-phase mode, coupled with a triple quadrupole mass spectrometer (SCIEX Api4000) operating in positive electrospray ionization mode. Detection was carried out in multiple reaction monitoring mode using 2 transitions per metabolite—1 for identification and 1 for quantification—along with retention time as a fixed parameter for metabolite identification (more details available in Supplementary Table 1). Absolute quantification of metabolites was based on calibration against reference standards. The limit of detection was defined as 3 to 5 times the blank signal for the parent and quantitative fragment ions. Metabolite quantification, quality control, and batch effect assessment are described in the Supplementary Methods and Supplementary Table 2; no significant batch effects were observed.

Of the 53 metabolites, 4 had <1% of values below the limit of detection and 1 had 7%; these values were imputed as half the lowest observed concentration for the corresponding metabolite [12,21]. Metabolite concentrations were log_2_-transformed to reduce skew, and extreme outliers [>3 standard deviation (SD) from the mean based on *z*-score scaling] were removed from individual metabolite models.

### Dietary intake

At each dietary collection period (repeated every 6 mo), participants were invited to complete 3 validated nonconsecutive 24-h dietary records (R24H), as previously described in detail [[22], [23], [24]]. In brief, all foods and beverages consumed were recorded on 2 weekdays and 1 weekend day, spaced over a 2-week period. Portion sizes were estimated using validated photographs [[22], [23], [24]] or entered manually. Daily intakes of energy, alcohol, macronutrients, and micronutrients were calculated from each R24H using the NutriNet-Santé food composition database, which includes over 3300 food items [25]. For composite dishes, individual ingredient amounts were estimated from standardized French recipes. Sodium intake reflected contributions from foods, cooking, and added table salt. All available R24Hs from inclusion up to the clinical visit (baseline) were used to estimate an average daily intake for 55 nutrients and 24 food groups.

### Statistical analysis

All statistical analyses were conducted in R (v4.4.2).

#### Metabolite patterns

To identify metabolite patterns, principal component (PC) analysis with varimax rotation was conducted on all metabolites [26]. The number of components retained was determined via scree plot inspection. Component scores were calculated for each participant and used as exposures in subsequent analyses.

#### Associations between metabolites and breast cancer risk

Multivariable Cox proportional hazard models were used to evaluate associations between individual metabolites, metabolite patterns, and breast cancer risk [27]. Participant age was used as the timescale to model time-to-event with inverse subcohort sampling probability weightings, assigning subcohort participants a weight of 1/(4200/17,603) [20]. Associations were reported as hazard ratios (HRs) with 95% confidence intervals (CIs).

Models were sequentially adjusted to examine covariate influence, guided by domain knowledge and prior literature [26,28]. Model 0 was unadjusted, aside from using age as the timescale, with follow-up starting at the age of blood collection. Model 1 (main model) included waist circumference (centimeter), height (centimeter), education level (less than high-school degree, ≤3 y of higher education, >3 y of higher education), family history of cancer (all cancers, immediate family; yes/no), prevalent health condition (recorded at baseline or within first year of follow-up, including type II diabetes [29,30], hypertension [31,32], and hypercholesterolemia [33]), smoking status (never, former, current) and smoking pack-years (continuous), alcohol intake (grams per day), physical activity (international physical activity questionnaire categories: low, medium, high), age at menarche (<12 y, 12–15 y, >15 y), number of biological children (continuous), age at birth of first child (no children, <20 y, 20–24 y, 25–34 y, ≥35 y), oral contraceptive use (ever; yes/no), menopausal status at baseline (pre/postmenopausal), and an interaction term between menopausal status and waist circumference (given that adult body fatness is inversely related to premenopausal breast cancer but a risk factor for postmenopausal breast cancer [3,34]). Missing data were minimal for covariates, with waist circumference missing for 3 participants (0.1%) and age at menopause for 1 participant (0.03%); missing values were imputed using the sample median.

Multiple sensitivity analyses were conducted. First, to account for multiple testing, *P* values from the main models were adjusted using the Benjamini-Hochberg false discovery rate method [35]. This adjustment was included as a sensitivity analysis to balance the risk of type I error (false positives) while avoiding overly conservative correction that could increase type II error (false negatives) and obscure moderate associations [36], reflecting the expectation that metabolites may act in coordinated networks where individual effects may be modest. Second, model 1–S1 included model 1 further adjusted for number of R24Hs (continuous), time from last R24H to baseline (years), total energy intake (kilocalorie per day, excluding alcohol), and 2 dietary pattern scores derived from PC analysis with varimax rotation on 24 food groups (more details available in Supplementary Figure 1). These scores reflected lower diet quality (e.g., higher fats/oils, sweets) and higher diet quality (e.g., more fruits, vegetables, whole grains). Third, model 1–S2 was model 1 excluding cases diagnosed <2 y from baseline to challenge reverse causality.

Subgroup analyses were conducted according to menopausal status. For participants who became menopausal during follow-up, menopause onset marked the censoring point in models of premenopausal breast cancer risk and the start point in models of postmenopausal breast cancer risk [28]. Model 1 was applied in both strata, omitting menopausal status and its interaction term, and recategorizing age of first birth (no children, <30 y, ≥30 y) to account for smaller sample sizes. Postmenopausal models (model 1–post) additionally adjusted for age at menopause (continuous) and menopausal hormone therapy use (ever; yes/no); premenopausal models did not (model 1–pre).

#### Exploring metabolite mediation of associations between diet and breast cancer risk

**FIGURE 2:**
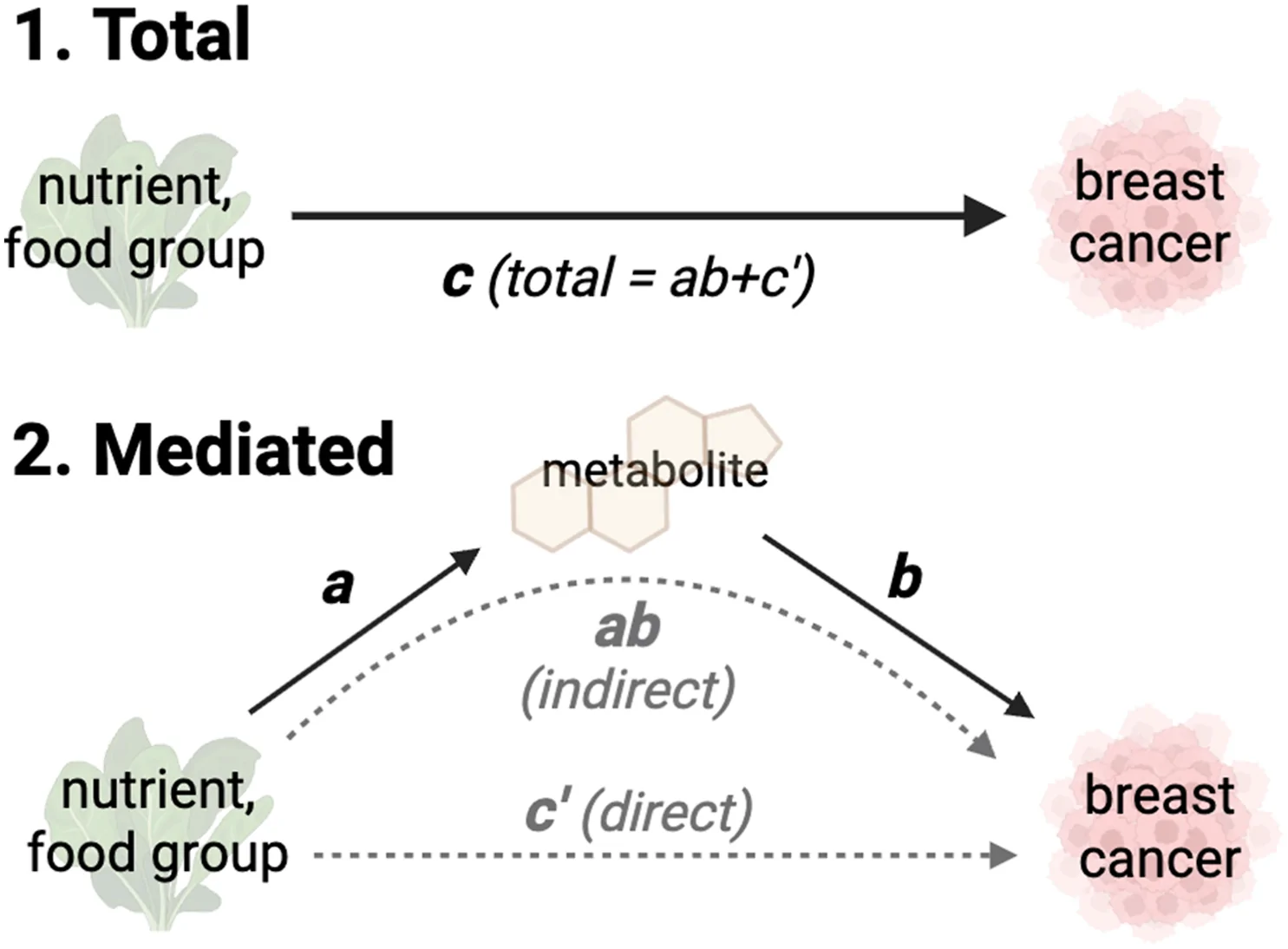
Mediation analysis showing total, direct, and indirect effects. The total effect of diet on breast cancer risk is shown by path *c*. The mediated pathway includes the association between diet and metabolite concentrations (path *a*), the association between metabolite concentrations and breast cancer risk (path *b*), and the resulting indirect effect (path *ab*). The direct effect of diet on breast cancer risk after accounting for the mediator is represented by path *c′*.

To explore dietary associations with metabolites, ElasticNet regression models incorporating both nutrients and foods were built for each metabolite found to be associated with breast cancer incidence (Figure 2, path *a*). ElasticNet combines L1 and L2 penalties, which allows correlated dietary variables to be retained and shrunk together, addressing the high multicollinearity common in nutrient and food data and capturing complex, interrelated dietary effects [37]. Covariates (as in model 1–S1, except excluding menopause interaction term, dietary pattern scores, and including age at baseline) were included as unpenalized terms. Models were tuned using 10-fold cross-validation repeated 10 times over 1100 α‒λ combinations, selecting parameters that minimized root mean squared error [26]. For each metabolite, selected dietary variables (nutrients/foods) were then jointly fit as predictors in a linear model to estimate their direct associations, reported as β coefficients with 95% CIs. Exponentiated β values (2^β^) indicated the percent change in metabolite concentration per 1-SD increase in dietary intake. A sensitivity analysis (model 1–S3) excluded participants with extreme energy intake outliers (<928 kcal/d or >2947 kcal/d, corresponding to the 1st and 99th percentiles).

Mediation analysis was conducted for significant diet–metabolite associations in linear models following ElasticNet regression, using the *Cmest* function [38]. The total (Figure 2, path *ab*+*c’*) and direct effect (path *c’*) of diet on breast cancer risk were estimated using a regression-based approach, adjusting for covariates as in model 1–S1, except excluding the menopause interaction term and including age at baseline. The indirect effect (path *ab*) was estimated using the imputation method, which calculates counterfactual outcomes by combining predicted values from the outcome and mediator models. Statistical significance was assessed using nonparametric bootstrapping (200 resamples initially; 1000 in final models), with *P* < 0.05 for the indirect effect indicating mediation.

## Results

### Sample characteristics

Characteristics of the 2993 participants are summarized in Table 1. Among the subcohort (*n* = 2762), the median age was 53.6 y (IQR = 21.1). Participants were followed for a median of 8.9 y (IQR = 3.7), totaling 23,595 person-years. Dietary intake was estimated from a median of 6 R24H per participant (range: 2–15), with the final R24H collected a median of 0.7 y before baseline (range: 0–0.47). During follow-up, 121 censoring events (106 other cancers, 15 deaths) occurred within the subcohort. Of the total 306 incident breast cancer events in the analytical sample (*n* = 306), 220 occurred >2 y after baseline.

**TABLE 1:** Characteristics of females with no prevalent cancer in the case-cohort study sample, NutriNet-Santé cohort, 2011‒2022 (*N* = 2993).

Characteristic	Subcohort (*n* = 2762)	Breast cancer cases^1^ (*n* = 306)
Age at baseline, y [median (Q1, Q3)]	53.6 (40.9, 62.0)	59.9 (51.3, 65.1)
Waist circumference, cm [median (Q1, Q3)]	78.0 (72.0, 86.0)	80.0 (75.0, 89.0)
Height, cm [mean (SD)]	162.5 (6.2)	162.4 (6.2)
Education level (%)		
Less than a high-school degree	554 (20.0)	67 (21.9)
<3 y of higher education	1325 (48.0)	156 (51.0)
≥3 y of higher education	883 (32.0)	83 (27.1)
Prevalent health condition,^2^ yes (%)	687 (24.9)	100 (32.7)
Family history of any cancer, yes (%)	1338 (48.4)	189 (61.8)
Smoking status (%)		
Never	1498 (54.2)	172 (56.2)
Former smoker	951 (34.4)	108 (35.3)
Current smoker	313 (11.3)	26 (8.5)
Number of cigarettes for smokers, pack-years [median (Q1, Q3)]	5.0 (1.0, 12.6)	6.0 (2.0, 15.0)
Physical activity, IPAQ categories (%)		
Low	501 (18.1)	56 (18.3)
Medium	1417 (51.3)	129 (42.2)
High	844 (30.6)	121 (39.5)
Energy intake without alcohol, kcal/d [mean (SD)]	1727.6 (407.4)	1720.1 (413.6)
Alcohol intake, g/d [median (Q1, Q3)]	3.3 (0, 9.1)	3.9 (0.7, 11.3)
Age at menarche (%)		
≥16 y (late)^3^	134 (4.9)	11 (3.6)
12‒15 y	2112 (76.5)	239 (78.1)
<12 y (early)	516 (18.7)	56 (18.3)
Number of biological children [median (Q1, Q3)]	2.0 (0, 2.0)	2.0 (1.0, 3.0)
Age at first birth (%)		
≥35 y	111 (4.0)	14 (4.6)
25–34 y	1145 (41.5)	148 (48.4)
20–24 y	637 (23.1)	81 (26.5)
<20 y	56 (2.0)	9 (2.9)
No children	813 (29.4)	54 (17.6)
Postmenopausal at baseline, yes (%)	1589 (57.5)	231 (75.5)
Age at menopause, y [median (Q1, Q3)]	51.0 (50.0, 53.0)	51.0 (50.0, 54.0)
Ever use of contraceptives, yes (%)	609 (22.0)	34 (11.1)
Ever use of hormone therapy, yes (%)	468 (16.9)	85 (27.8)

Abbreviations: IPAQ, international physical activity questionnaire; SD, standard deviation; Q1, Q3, quartile.

1 Of the 306 breast cancer cases, 75 were part of the subcohort.

2 Health condition prevalent at baseline or diagnosed within 1 y follow-up, included diabetes type 2 (*n* = 58 in the subcohort, *n* = 6 in breast cancer cases), hypertension (*n* = 374 subcohort, *n* = 58 cases), hypercholesterolemia (*n* = 446 subcohort, *n* = 66 cases).

3 This group also contained 4 females who never had a menstrual period.

### Metabolite associations and breast cancer risk

**FIGURE 3:**
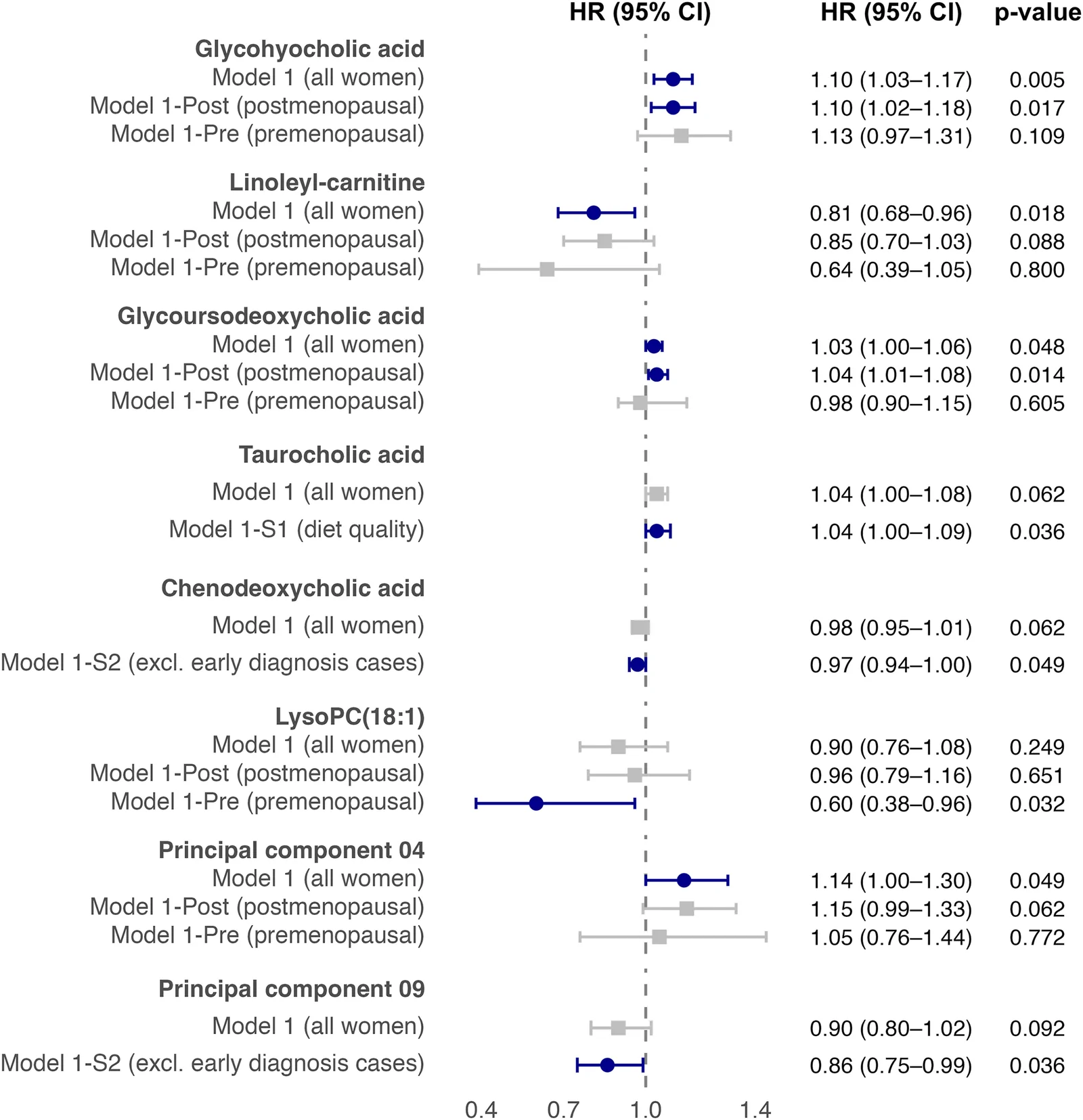
Log_2_-transformed metabolites and metabolite patterns associated with breast cancer risk reported from Cox proportional hazards models in the NutriNet-Santé case-cohort study, *N* = 2993, 2011–2022. Blue circles distinguish significant associations (*P* < 0.05) from nonsignificant (gray squares). PC04 was primarily loaded by glycocholic acid (loading = 0.83), glycochenodeoxycholic acid (loading = 0.78), glycodeoxycholic acid (loading = 0.67), glycoursodeoxycholic acid (loading = 0.62), taurocholic acid (loading = 0.61), glycohyocholic acid (loading = 0.41) (Supplementary Figure 2). PC09 was driven by cholic acid (loading = 0.66), chenodeoxycholic acid (loading = 0.62), and deoxycholic acid (loading = 0.61) (Supplementary Figure 3). Model 1 (overall sample; 306 breast cancer cases; 23,595 person-years) was adjusted for waist circumference (continuous), height (continuous), education level (less than high-school degree, ≤3 y of higher education, >3 y of higher education), family history of cancer (all types, immediate family; yes/no), prevalent health condition at baseline or diagnosed within 1 y follow-up (includes type II diabetes, hypertension, hypercholesterolemia), smoking status (never, former, current) and smoking pack-years (continuous), alcohol intake (grams per day), physical activity (IPAQ categories: low, medium, high), age at menarche (<12 y, 12–15 y, >15 y), number of biological children (continuous), age at first birth (no children, <20 y, 20–24 y, 25–34 y, ≥35 y), contraceptive use (ever; yes/no), menopausal status at baseline (pre/postmenopausal), and an interaction term between menopausal status and waist circumference. Model 1–post (postmenopausal stratum; 261 cases; 16,743 person-years) and model 1–pre (premenopausal stratum; 45 cases; 9807 person-years) were adjusted as in model 1, except excluding menopause status and interaction term, and recategorizing age of first birth (no children, <30 y, ≥30 y) to account for smaller sample sizes. Model 1–post additionally adjusted for age at menopause and menopause hormone therapy use. CI, confidence interval; HR, hazard ratio; IPAQ, international physical activity questionnaire; LysoPC, lysophosphatidylcholine; PC, principal component.

In the main model (model 1; Figure 3), glycohyocholic acid (HR: 1.10; 95% CI: 1.03, 1.17) and glycoursodeoxycholic acid (HR: 1.03; 95% CI: 1.00, 1.06) were associated with increased breast cancer risk, whereas linoleyl-carnitine was inversely associated (HR: 0.81; 95% CI: 0.68, 0.96). Similar effect sizes across unadjusted (model 0) and adjusted models (model 1) suggested these associations were independent of demographic, reproductive, and lifestyle factors (Supplementary Table 3).

In sensitivity analyses, glycohyocholic acid remained significant after multiple testing correction (false discovery rate <0.25). Both glycohyocholic acid and linoleyl-carnitine remained significant after further adjustment for dietary patterns (model 1‒S1) and after excluding breast cancer cases diagnosed within 2 y of follow-up (model 1-S2). Model 1–S1 additionally identified a positive association for taurocholic acid (TCA) (HR: 1.04; 95% CI: 1.00, 1.09), whereas model 1–S2 revealed an inverse association for chenodeoxycholic acid (HR: 0.97; 95% CI: 0.94, 1.00; Figure 3).

In stratified analysis, findings for postmenopausal females (261 cases; 16,743 person-years; model 1–post) were similar to the overall results (Supplementary Table 3). Among premenopausal females (45 cases; 9807 person-years; model 1–pre), only lysophosphatidylcholine (lysoPC) (18:1) was significant, showing an inverse association (HR: 0.60; 95% CI: 0.38, 0.96) not observed in other groups (Figure 3).

Metabolite pattern analysis retained 11 PCs (Supplementary Table 4). PC04 was associated with increased breast cancer risk (HR: 1.14; 95% CI: 1.00, 1.30; model 1; Figure 3). PC04 explained 5.8% of the variance in metabolite concentrations and was driven by glycocholic acid (loading = 0.83), glycochenodeoxycholic acid (loading = 0.78), glycodeoxycholic acid (loading = 0.67), glycoursodeoxycholic acid (loading = 0.62), TCA (loading = 0.61), glycohyocholic acid (loading = 0.41) (Supplementary Figure 2). Excluding early diagnosed cases (<2 y follow-up; model 1–S2), we additionally identified PC09 as inversely associated with breast cancer risk (HR: 0.86; 95% CI: 0.75, 0.99). PC09 explained 3.5% of variance and was driven by cholic acid (loading = 0.66), chenodeoxycholic acid (loading = 0.62), and deoxycholic acid (loading = 0.61) (Supplementary Figure 3).

### Association between dietary intakes and breast cancer–related metabolites

Although adjustment for overall diet quality patterns in sensitivity analyses (model 1‒S1 above) did not change metabolite–breast cancer associations, we examined whether these metabolites were associated with individual nutrients and food groups. ElasticNet selected between 1 and 30 dietary variables per metabolite (Supplementary Table 5), with Table 2 reporting diet–metabolite associations that remained significant in subsequent linear models of all selected dietary variables jointly fit per metabolite.

**TABLE 2:** Significant diet–metabolite associations determined by linear models of ElasticNet-selected dietary variables and metabolites associated with breast cancer incidence, NutriNet-Santé case-cohort study, *N* = 2993, 2011‒2022.

Metabolite	Dietary variables				
	Numberselected^1^	Significant in models^2^ (diet SD unit/day)	β (95% CI)	2ˆβ^3^ (%)	*P* value
Glycohyocholic acid	12	Vitamin K (125.1 *μ*g/d)	‒0.12 (‒0.20, ‒0.04)	‒8.4	0.003
		Processed meats (23.3 g/d)	‒0.09 (‒0.16, ‒0.02)	‒5.9	0.017
		Plant lipids (14.9 g/d)	0.11 (0.01, 0.20)	7.6	0.028
		Refined grains (70.7 g/d)	0.09 (0.00, 0.18)	6.5	0.046
Linoleyl-carnitine	30	Breakfast cereals (13.5 g/d)	0.05 (0.02, 0.08)	3.6	0.0004
		Milk and soft cheeses (118.1 g/d)	‒0.05 (‒0.08, ‒0.02)	‒3.2	0.002
		Potatoes and tubers (39.6 g/d)	0.04 (0.01, 0.07)	2.8	0.003
		Yogurt (70.9 g/d)	‒0.04 (‒0.07, ‒0.01)	‒2.8	0.004
		Cakes and biscuits (42.4 g/d)	0.05 (0.01, 0.09)	3.7	0.007
		Vitamin E (4.3 mg/d)	0.05 (0.01, 0.10)	3.6	0.028
Glycoursodeoxycholic acid	22	Oilseeds (11.2 g/d)	‒0.36 (‒0.53, ‒0.18)	‒22.0	6.3 × 10^‒05^
		Vitamin E (4.3 mg/d)	0.42 (0.20, 0.64)	33.8	0.0001
		Docosapentaenoic acid (0.19 g/d)	0.20 (0.02, 0.39)	15.1	0.033
		Complex carbohydrates (32.8 g/d)	0.27 (0.02, 0.52)	20.5	0.034
		DHA (0.20 g/d)	‒0.21 (‒0.41, ‒0.01)	‒13.7	0.037
Taurocholic acid	4	Soups and broths (45.6 g/d)	0.17 (0.07, 0.27)	12.5	0.001
		Breakfast cereals (13.5 g/d)	0.12 (0.02, 0.22)	8.9	0.017
Chenodeoxycholic acid	10	β-carotene (2619.1 *μ*g/d)	0.26 (0.10, 0.42)	20.0	0.001
		Oilseeds (11.2 g/d)	‒0.28 (‒0.45, ‒0.11)	‒17.5	0.001
		Milk and soft cheeses (118.1 g/d)	‒0.19 (‒0.35, ‒0.03)	‒12.4	0.018
LysoPC (18:1)	17	Linoleic acid (4.2 g/d)	‒0.06 (‒0.11, ‒0.01)	4.2	0.011
		Potatoes and tubers (39.6 g/d)	0.03 (0.01, 0.06)	2.3	0.020
		Cakes and biscuits (42.4 g/d)	0.04 (0.00, 0.07)	2.6	0.024
		Caffeine (162.1 mg/d)	0.03 (0.00, 0.06)	2.4	0.028
Principal Component 04	22	Breakfast cereals (13.5 g/d)	0.07 (0.03, 0.12)	5.1	0.002
		Vitamin K (125.1 *μ*g/d)	‒0.07 (‒0.11, ‒0.02)	‒4.5	0.002
		Processed meats (23.3 g/d)	‒0.04 (‒0.09, 0.00)	‒3.0	0.038
Principal component 09	1	Oilseeds (11.2 g/d)	‒0.07 (‒0.10, ‒0.03)	‒4.6	0.0003

Abbreviations: CI, confidence interval; IPAQ, international physical activity questionnaire; LysoPC, lysophosphatidylcholine; R24H, 24-h dietary record; SD, standard deviation.

1 Number selected refers to the number of dietary variables having nonzero coefficients in ElasticNet models for each metabolite associated with breast cancer incidence (Supplementary Table 5).

2 Linear regression models of all ElasticNet-selected dietary variables to metabolites were adjusted for age at baseline (continuous), waist circumference (continuous), height (continuous), education level (less than high-school degree, ≤3 y of higher education, >3 y of higher education), family history of cancer (all types, immediate family; yes/no), prevalent health condition at baseline or diagnosed within 1 y follow-up (includes type II diabetes, hypertension, hypercholesterolemia), smoking status (never, former, current) and smoking pack-years (continuous), alcohol intake (gram per day), physical activity (IPAQ categories: low, medium, high), age at menarche (<12 y, 12–15 y, >15 y), number of biological children (continuous), age at first birth (no children, <20 y, 20–24 y, 25–34 y, ≥35 y), contraceptive use (ever; yes/no), menopausal status at baseline (pre/postmenopausal), number of R24H (continuous), time from last R24H to baseline (continuous), and energy intake (kilocalories per day excluding alcohol).

3 To facilitate interpretation, the coefficient (β) was exponentiated (2^β^) to reverse the log_2_ transformation applied to the metabolite concentrations. The exponentiated coefficient (2^β^) represents the direct change in metabolite concentrations for a 1 SD increase in the dietary variable, whereas the raw coefficient corresponds to the fold change.

For glycohyocholic acid (*n* = 12 ElasticNet-selected dietary variables), 4 were significant in linear regression: vitamin K and processed meats were inversely associated with metabolite concentrations, and plant lipids and refined grains were positively associated. Vitamin K showed the strongest association, with a 125.1 *μ*g/d increase linked to an 8.4% decrease in glycohyocholic acid (β: ‒0.12; 95% CI: ‒0.20, ‒0.04). Linoleyl-carnitine (*n* = 30) had 6 significant dietary associations, including inverse links with milk and soft cheeses and yogurt and positive associations with breakfast cereals, potatoes and tubers, cakes and biscuits, and vitamin E; effect sizes were modest (∼3% change in metabolite concentrations). Glycoursodeoxycholic acid (*n* = 22) had 5 significant dietary associations: oilseeds and DHA linked to lower metabolite concentrations and vitamin E, docosapentaenoic acid, and complex carbohydrates to increased levels; effect sizes were largest among all metabolites (15%–34%). TCA (*n* = 4) and chenodeoxycholic acid (*n* = 10) had fewer significant dietary links, with soups and broths, β-carotene, and oilseeds among notable factors. LysoPC (18:1) (*n* = 17) showed positive associations with potatoes and tubers, cakes and biscuits, and caffeine, and an inverse association with linoleic acid. PC04 (*n* = 22) was positively associated with breakfast cereals and inversely associated with vitamin K and processed meats. PC09 (*n* = 1) showed an inverse association with oilseeds.

Sensitivity analysis excluding participants in the bottom and top percentiles of energy intake showed generally consistent results with the above findings, except for lysoPC (18:1) and PC09, which displayed marked differences in the number of selected dietary variables (Supplementary Table 5).

### Metabolite mediation in diet–breast cancer associations (paths *ab* and *c’*)

**FIGURE 4:**
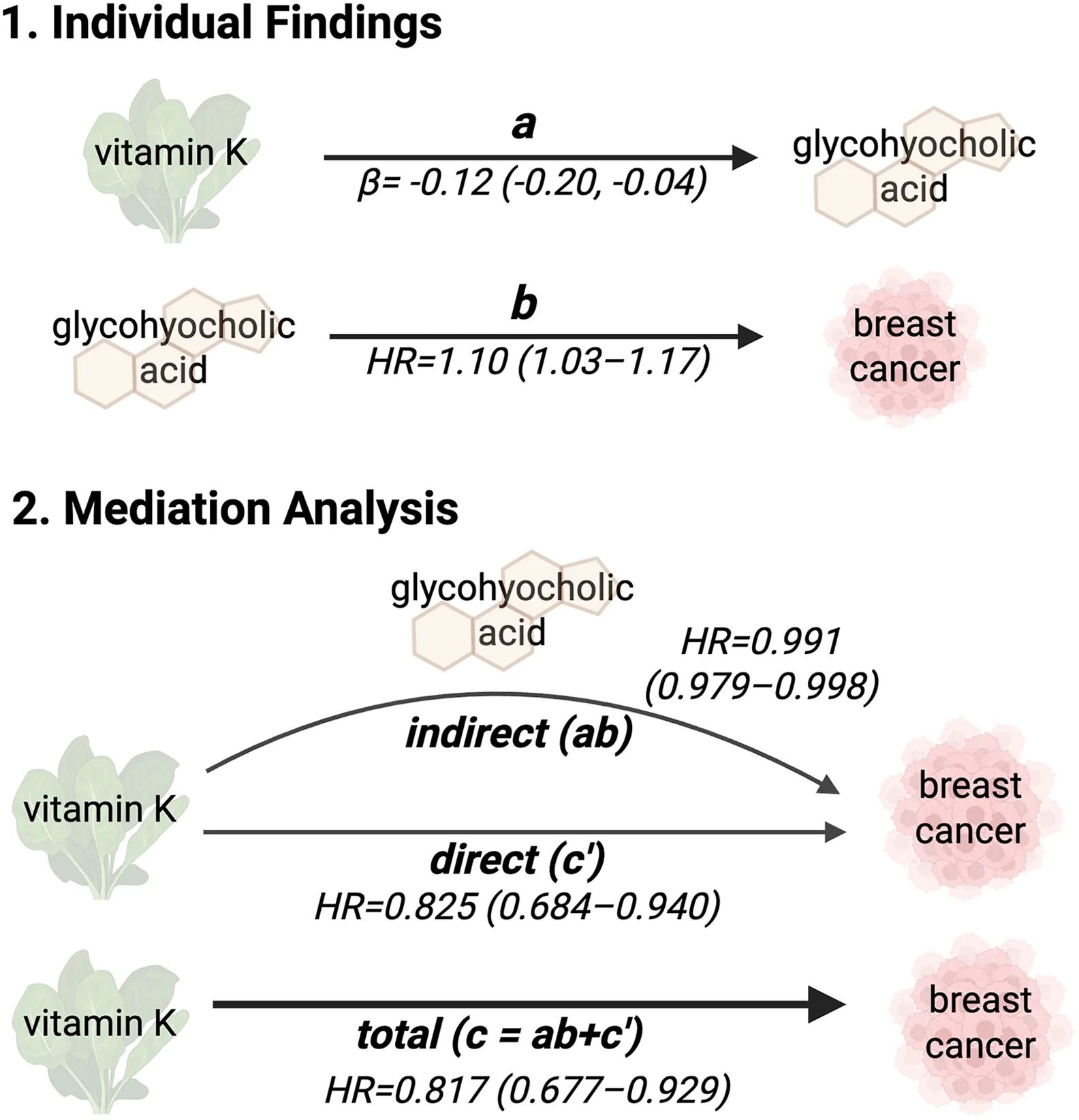
Example of results for the vitamin K–glycohyocholic acid–breast cancer pathway, showing individual associations (diet–metabolite and metabolite–breast cancer) and mediation analysis results. HR, hazard ratio.

All significant diet–metabolite relationships were further examined using mediation analysis (Supplementary Table 6). Among dietary variables, only vitamin K showed a significant direct association with breast cancer (HR: 0.83; 95% CI: 0.68, 0.94; Figure 4 path *c’*). The vitamin K–associated metabolite (glycohyocholic acid) and metabolite pattern (PC04) both demonstrated significant indirect effects. The indirect effect via glycohyocholic acid (HR: 0.99; 95% CI: 0.98, 1.00; Figure 4 path *ab*) accounted for 4.2% of the total effect, resulting in a slightly stronger total effect estimate (HR: 0.82; 95% CI: 0.68, 0.93; Figure 4 path *ab+c’*). The indirect effect via PC04 (HR: 0.99; 95% CI: 0.98, 1.00) accounted for 4.1% of the total effect, also yielding a slightly stronger total effect (HR: 0.82; 95% CI: 0.70, 0.94). These findings suggest that vitamin K’s association with breast cancer was primarily direct, with small but significant portions mediated through glycohyocholic acid and PC04.

## Discussion

In this case-cohort analysis of incident breast cancer nested within the NutriNet-Santé cohort, we identified novel associations between conjugated bile acids and breast cancer risk within an integrative framework linking diet, metabolites, and disease. Specifically, higher concentrations of glycohyocholic acid, glycoursodeoxycholic acid, TCA, and a metabolite pattern enriched in conjugated bile acids (PC04) were associated with increased breast cancer incidence. In contrast, linoleyl-carnitine, chenodeoxycholic acid, and a pattern enriched in unconjugated bile acids were inversely associated with risk. Among premenopausal females, lysoPC (18:1) was associated with a lower incidence of breast cancer.

PC04 was enriched in multiple glycine-conjugated bile acids, including glycocholic acid, glycochenodeoxycholic acid, glycodeoxycholic acid, glycoursodeoxycholic acid, glycohyocholic acid, and TCA (taurine-conjugated). Conjugated bile acids—particularly glycohyocholic acid—have not been widely reported in prospective breast cancer metabolomics studies, but the few available reports are broadly consistent with our findings, showing higher levels in breast cancer cases [39,40]. Several conjugated bile acids, particularly glycocholic acid and TCA, can activate Sphingosine-1-Phosphate Receptor 2–Extracellular Signal-Regulated Kinase/Protein Kinase B–Cyclooxygenase-2 signaling linked to inflammation and migration [41,42]. Conjugates such as TCA can also activate Takeda G protein-coupled Receptor 5, influencing cell proliferation and epithelial metabolic signaling [41,43]. TCA may promote risk indirectly through sulfate-reducing bacteria metabolizing taurine to hydrogen sulfide, a pro-inflammatory and genotoxic compound at high concentrations [44]. In contrast, glycoursodeoxycholic acid (a tertiary bile acid) has been reported to exert protective effects via gut microbial bile acid modulation [45]. Taken together, these findings suggest that conjugated bile acids may interact with signaling pathways relevant to inflammation and tumor biology, although their mechanistic roles appear complex and context dependent.

PC09, enriched in unconjugated primary bile acids (cholic acid, chenodeoxycholic acid) and the secondary bile acid deoxycholic acid, may reflect lower hepatic conjugation or greater microbial deconjugation. These unconjugated bile acids are stronger Farsenoid X Receptor (FXR) agonists than conjugated forms; FXR activation in breast cancer models suppresses proliferation, opposes estrogen receptor signaling, and reduces migration [43,46,47]. The opposing associations of PC04 and PC09 highlight the potential relevance of bile acid composition and conjugation status, which may be influenced by gut microbial bile salt hydrolase activity. These findings extend prior work by suggesting that not only bile acid concentrations, but their conjugation status, may be relevant to breast cancer risk.

Linoleyl-carnitine was inversely associated with breast cancer incidence, consistent with prior findings [12]. This long-chain acylcarnitine facilitates mitochondrial β-oxidation of linoleic acid, potentially supporting a more oxidative, antitumor metabolic state [48,49], modulating inflammation and estrogen signaling [50], and possibly linking gut microbiota to a gut–liver–hormone axis in breast cancer [50].

Metabolite associations differed by menopausal status, supporting established differences in hormonal environment, risk factors, and etiology [51]. LysoPC (18:1) was inversely associated with premenopausal breast cancer, consistent with previously reported findings in all females [13]. This lysophospholipid belongs to the lysoPC class, which regulates membrane signaling and immune responses [52] and of which reduced levels have been reported across cancers [9,13,53]. Mechanisms remain uncertain, but lysoPCs may influence inflammatory signaling through G protein-coupled receptors and toll-like receptors [52,54], with estradiol potentially modulating these effects [55].

A brief review identified 13 prospective metabolomics studies on breast cancer: 9 nested case-controls [[9], [10], [11], [12],^14^,^15^[9-12,14,15,[56], [57], [58], [59], 2 case-cohorts [13,53], and 1 cohort study [60]—spanning 8 cohorts, with overlap in PLCO[15,57], EPIC [9,53], SU.VI.MAX [11,56], CPS [12,13], and NHS[14,59]. Most studies applied liquid chromatography–mass spectrometry for their metabolite measurement, and 3 [10,56,60] applied proton nuclear magnetic resonance, using a mix of untargeted (*n* = 5) and targeted (*n* = 8, ranging from 3 to 1275 metabolites) approaches. However, these studies have differed in metabolite coverage, analytic strategies, and reporting practices, limiting comparability across findings.

Of the 53 metabolites examined in our current study, only 12 had previously reported associations: 5 linked to increased risk (leucine [10,60], hypoxanthine [10], tryptophane [11], phenylalanine [11], acetyl-carnitine [9]); 6 to decreased risk (sphinganine, sphinganine-1P, lysoPC (18:1) and lysoPE (18:1) [13], lysoPE (18:0) [13,53], linoleyl-carnitine [12]); and arginine showed mixed results [9,56]. Of these 12 associations, 2 were replicated in our study [linoleyl-carnitine, lysoPC (18:1)] while the remaining were not observed. Rather than reflecting a lack of reproducibility alone, these differences likely arise from heterogeneity in study populations, metabolite panels, and analytic approaches.

Importantly, prior studies have largely evaluated metabolite–disease associations without integrating upstream dietary exposures. The only prospective study to explicitly examine diet-related metabolites [15] focused on metabolites preselected on dietary correlations, with minimal overlap—only 2 metabolites—between their diet-correlated panel and the 53 metabolites examined in the current study. In contrast, this study applied a metabolite-first approach, identifying metabolites associated with breast cancer risk before evaluating their dietary predictors using multivariate methods and assessing their potential mediating role. This integrative framework enables a more direct assessment of whether metabolites lie along the pathway linking diet to breast cancer, rather than assuming diet relevance a priori.

The second aim of this study extended beyond prior metabolomics analyses by evaluating how diet may influence metabolite-related pathways in breast cancer and identified vitamin K intake to be inversely associated with glycohyocholic acid and PC04, with each SD increase (125.1 *μ*g/d) in vitamin K linked to 8.4% and 4.5% reductions, respectively. The moderate loading of glycohyocholic acid within PC04 (0.41) suggests it is not the dominant driver of the pattern—both may reflect broader pathway-level variation in conjugated bile acid metabolism as they are essential for the absorption of fat-soluble vitamins such as vitamin K.

Mediation analysis indicated that vitamin K intake was inversely associated with breast cancer risk (HR = 0.825), with reductions in glycohyocholic acid and PC04 each modestly but significantly explaining ∼4% of this association. This represents a novel application of mediation analyses in prospective breast cancer metabolomics, providing evidence that these metabolites may partially lie along the pathway linking diet to disease. Vitamin K_1_ (phylloquinone), derived mainly from leafy green vegetables, functions primarily in hepatic coagulation, whereas vitamin K_2_ (menaquinones), obtained from fermented foods and animal products, is more bioavailable in extrahepatic tissues and has a longer half-life [61]. Although breast cancer mechanisms remain unclear, experimental evidence for vitamin K_2_ suggests antiproliferative and antimetastatic effects, including modulation of inflammatory signaling and angiogenesis [62,63]. A Mendelian randomization study further suggests a protective role of microbial menaquinol-8 biosynthesis (pathway PWY-6263) [64]. Similarly, a case-control study of postmenopausal Estrogen Receptor-positive breast cancer found lower abundance of microbial menaquinol-8 biosynthesis pathways in cases compared to controls [40]. However, observational vitamin K studies remain inconsistent and vary by subtype [62,65]. Given these distinctions, the observed association may more strongly reflect vitamin K_2_ activity or sources, particularly considering the microbial contribution to menaquinone production.

Strengths of this study include the prospective case-cohort design, which enhances temporal inference through the use of incident cases after blood draw and improves generalizability via random sampling of the subcohort from the source population. Sensitivity analyses excluding breast cancer cases within 2 y of follow-up (and thus likely prevalent but undetected disease at baseline) confirmed the robustness of associations. Detailed dietary data and extensive covariate adjustment allowed for a comprehensive assessment of dietary influences and confounders. The integration of dietary, metabolomic, and disease data within a unified analytical framework enhanced the interpretation of potential etiologic pathways. Limitations include the use of a limited, noncancer and non-diet-specific metabolomics panel, lack of information on molecular subtypes of breast cancer, and single-time-point metabolite measurements, though prior studies suggest stability over time [66,67]. Modest effect sizes and the possibility of residual confounding highlight the need for further research to validate findings. Although the cohort included females of all ages, most were postmenopausal, so findings are likely most relevant to this group and to HR+ breast cancer, which accounts for ∼75% of cases [68]. Studies in younger females, males, or populations with different dietary patterns will be needed to assess broader applicability.

In conclusion, this study provides novel evidence linking conjugated bile acids to breast cancer risk within an integrative diet–metabolite–disease framework. Conjugated bile acids were positively associated with breast cancer risk, whereas unconjugated bile acids were inversely associated, suggesting a potential role for bile acid conjugation in carcinogenesis. Glycohyocholic acid emerged as a novel finding both independently and within the conjugated bile acid pattern. Linoleyl-carnitine and lysoPC (18:1) were associated with lower breast cancer incidence, with associations varying by menopausal status. Several dietary factors were associated with these metabolites, and vitamin K intake showed an inverse association with breast cancer risk, a small portion of which was mediated through reductions in conjugated bile acids and glycohyocholic acid. These findings highlight the potential for diet to influence breast cancer risk through metabolic pathways. Future studies using harmonized methods that integrate metabolomics and dietary data are needed to clarify the interplay between diet, metabolism, and breast cancer risk, and to reproduce these findings.

## Author contributions

The authors’ responsibilities were as follows – AHL, RAM, MT, MD-T: designed research; BS, ND, PG, SH, LB: conducted research; CD, OG: processed metabolites; AHL: analyzed data; AHL, RAM, MT, MD-T: wrote the article; AHL: had primary responsibility for final content; and all authors: read and approved the final manuscript.

## Data availability

Researchers from public institutions can submit a request to have access to the data for strict reproducibility analysis (systematically accepted) or for a new collaboration by providing information about their institution and a brief description of the project to collaboration@etude-nutrinet-sante.fr. All requests will be reviewed by the steering committee of the NutriNet-Santé study. If the collaboration is approved, a data access agreement will be required, and any necessary authorizations from the relevant administrative authorities may be needed. In compliance with existing regulations, no personally identifiable data will be accessible.

## Funding

This project was supported by the France-Canada Research Fund, Mitacs Globalink Research Award, and the Canadian Institutes of Health Research Doctoral Award (to AHL). The NutriNet-Santé study received support from the following public institutions: Ministère de la Santé, Santé Publique France, Institut National de la Santé et de la Recherche Médicale (INSERM), Institut National de la Recherche pour l’agriculture, l’alimentation et l’environnement (INRAE), Conservatoire National des Arts et Métiers (CNAM), and Université Sorbonne Paris Nord. Metabolomics analyses were funded by the European Research Council under the European Union’s Horizon 2020 research and innovation program (grant agreement number 864219, ADDITIVES). The funding sources had no role in the study design, the collection, analysis, or interpretation of data, the writing of the report, or the decision to submit the manuscript for publication.

## Declaration of generative AI and AI-assisted technologies in the writing process

The author(s) declare that no generative AI or AI-assisted technologies were used in the writing of this manuscript.

## Conflict of interest

The authors report no conflicts of interest.
