# Supplementary material for: Plasma metabolites, dietary intakes, and breast cancer incidence: a prospective case-cohort study in NutriNet-Santé
Source: Am J Clin Nutr. 2026 May 23;124(1):101342. doi: 10.1016/j.ajcnut.2026.101342 (PMC13494087; doi:10.1016/j.ajcnut.2026.101342)
Supplement: Supplementary file 1 [file mmc1.pdf]

Supplementary Information

Plasma metabolites, dietary intakes, and breast cancer incidence: a prospective case-cohort study in NutriNet-Santé

Authors

Ashley H. Larnder[1], Rachel A. Murphy[1,2], Bernard Srour[3,4], Nicolas Déchamp[3], Pilar Galan[3,4], Serge Hercberg[3,4], Laurent Bourhis[3], Caridad Díaz[5], Olga Genilloud[5], Mathilde Touvier[3,4*], Mélanie Deschasaux-Tanguy[3,4*].

Affiliations

*co-last authors

[1] School of Population and Public Health, University of British Columbia, Vancouver, Canada

[2] Population Health Sciences, BC Cancer, Vancouver, Canada

[3] Université Sorbonne Paris Nord and Université Paris Cité, INSERM, INRAE, CNAM, Centre of Research in Epidemiology and StatisticS (CRESS), Nutritional Epidemiology Research Team (EREN), F-93017 Bobigny, France

[4] NACRe network, https://www.nacrenetwork.eu, Jouy-en-Josas, France

[5] Centro de Excelencia en Investigación de Medicamentos Innovadores en Andalucía, Avda. del Conocimiento 34, 18016 Armilla (Granada), Spain.

Supplementary Methods

Metabolite Quantification and Quality Control

Lower and upper limits of quantification (LLOQ, ULOQ) were established for each metabolite individually (**Supplementary Table 2**). The LLOQ was defined as 10× the blank signal, and the ULOQ as the concentration at which the signal began to saturate. The limit of detection (LOD) was defined as 3–5× the blank signal for the parent and quantitative fragment ions. Calibration curves were constructed with 8½ dilution points for each metabolite and validated on two separate days following FDA guidelines (USDHHS, FDA, 2018).

For 65% of metabolites, all samples were quantified within the linear range. For the remainder, most samples were within the linear range, and values outside the range were extrapolated carefully using the calibration curve, respecting the LLOQ and ULOQ (**Supplementary Table 2**). Values below the LOD were reported as "below detection limit."

Samples were analyzed in 13 batches with approximately equal numbers per batch. Each batch included calibration curves at the beginning and end, and three QC samples every 30 cohort samples, spanning low, medium, and high concentrations. Calibration curves achieved R^2 ≥0.99 (≥0.98 in rare cases). Reproducibility was assessed using intra- and inter-assay CVs, with acceptance criteria CV <15% and accuracy 80–120%. QC samples met these criteria for ≥67% of measurements.

Metabolite batch effects

To assess batch effects in the metabolomics data, each metabolite was modeled with a Cox regression including batch as a covariate, and the significance of the batch term was evaluated using Type III Wald tests. No significant batch effects were observed (mean p-value=0.71; range 0.54–0.88), suggesting that batch did not influence metabolite-breast cancer relationships (Supplementary Table 2).

Citation: USDHHS, FDA, CDER. Guidance for Industry: Bioanalytical Method Validation. U.S. Department of Health and Human Services, Food and Drug Administration, Center for Drug Evaluation and Research (CDER), 2018.

Supplementary Figures

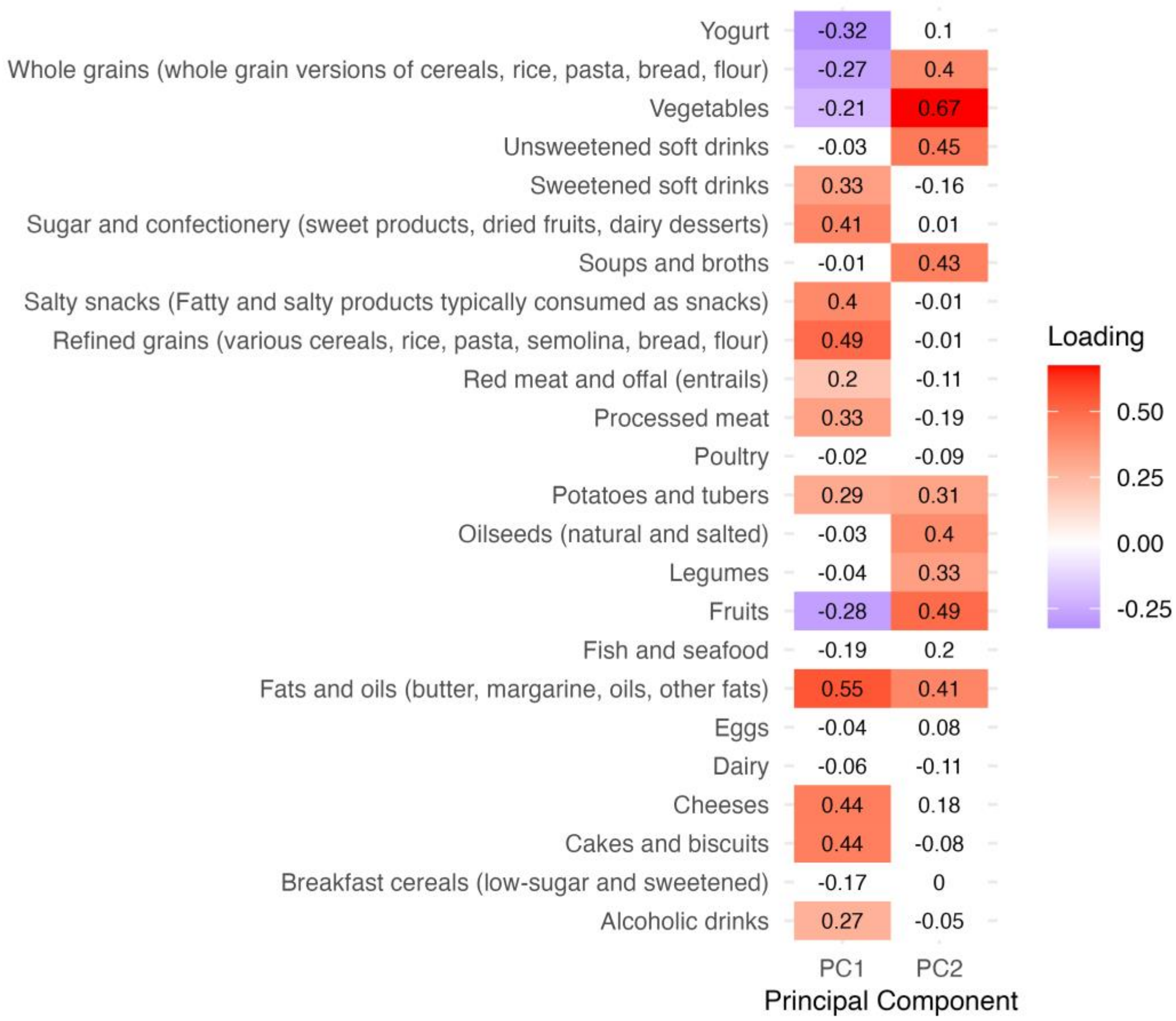

Supplementary Figure 1. Factor loadings plot for principal component analysis with orthogonal (varimax) rotation on two factors based on 24 food categories in the NutriNet-Santé case-cohort study, N=2993, 2011–2022. The first two principal components were retained, as they explained a large proportion of variance and captured distinct dietary patterns: PC1 reflected a lower-quality diet including higher intake of higher fats and oils, refined grains, sugary products; PC2 reflected a higher-quality diet with higher intake of vegetables, fruits, unsweetened beverages.

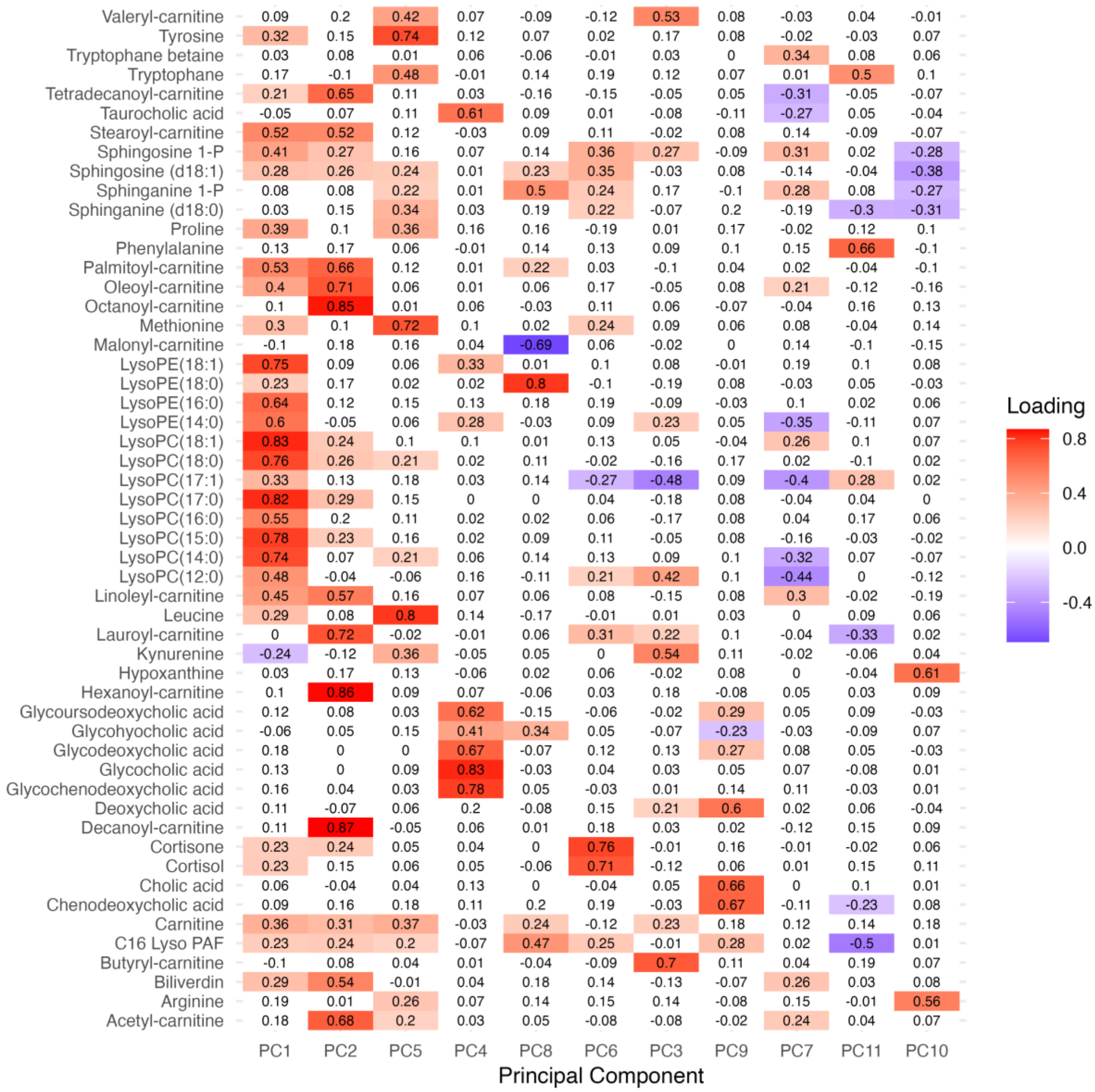

Supplementary Figure 2. Factor loadings plot for principal component analysis with orthogonal (varimax) rotation on 11 factors for the 53 log_2-transformed metabolites in the in the NutriNet-Santé case-cohort study, N=2993, 2011–2022.

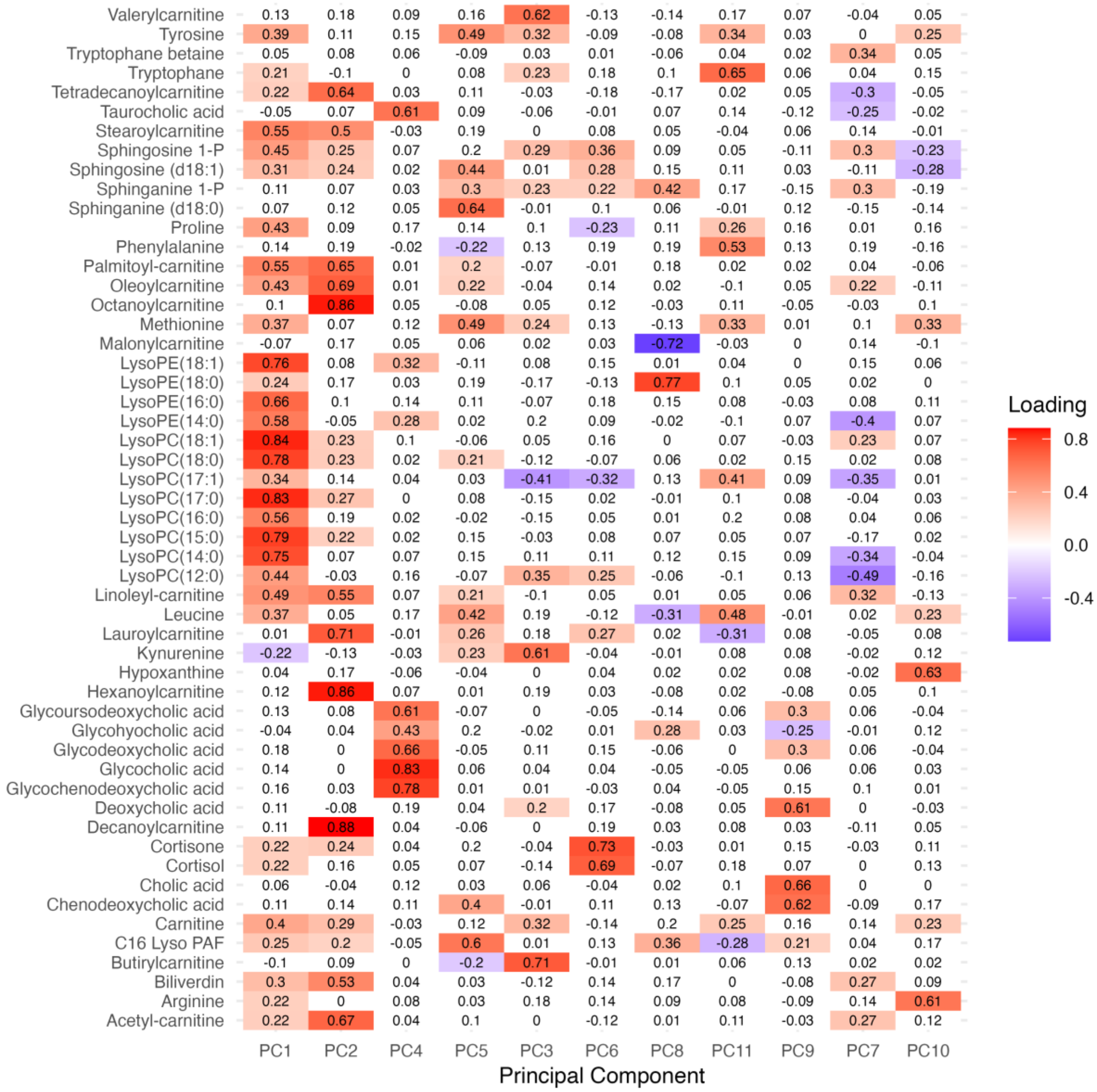

Supplementary Figure 3. Factor loadings plot for principal component analysis with orthogonal (varimax) rotation on 11 factors for the 53 log_2-transformed metabolites after removing cases who were diagnosed within the first two years of follow-up (n=86) in the NutriNet-Santé case-cohort study, N=2993, 2011–2022.

Supplementary Tables

Supplementary Table 1. Summary of mass spectrometry and liquid chromatography conditions

**Mass spectrometey conditions**	
Decluster Potential (Volts)	55-80
Entrance Potential (Volts)	10
Collision Energy (Volts)	13-93
Curtain Gas (psi)	15
Ion Source Gas 1 (psi)	45
Ion Source Gas 1 (psi)	50
IonSpray Voltage (Volts)	5500
Temperature (ºC)	480
**Liquid chromatography conditions**	
Mobile Phase A	Water/acetonitrile (90/10) / 0.01% formic acid
Mobile Phase B	Acetonitrile/water (90/10) / 0.01% formic acid
Flow Rate (µl/minute)	250
Gradiente	0.0-0.5 min 1% MP B; 0.5-6.1 min 99% MP B; 6.1-8.2 min 99% MP B; 8.2-8.3 min 1%B
Run Time (minutes)	9.5
Column Type	Atlantis T3 C18 (2.1 mm x 50 mm, 3 µm) from Waters
Column Oven Temperature (ºC)	27
Injection Volume (µl)	3

MP: Mobile phase

Supplementary Table 2. Metabolite measurement characteristics (limits of quantification and linear calibration ranges) and evaluation of metabolite batch effects in Cox proportional hazard models of breast cancer incidence, NutriNet-Santé nested case-cohort, 2011-2022, N=2993.

		Limits of Quantification		Calibration Curve	Batch Effects	
**Metabolite**	**Metabolite Class**	**LLOQ (nmol/L)**	**ULOQ (nmol/L)**	**Linear range (nmol/L)**	**Chisq**	**P-value**
Arginine	Amino Acids	69	126300	70 – 8955	9.2	0.68
Kynurenine	Amino Acids	34	37460	59 – 7493	8.9	0.71
Leucine	Amino Acids	30	60980	93 – 11890	9.1	0.69
Methionine	Amino Acids	42	57640	45 – 5710	9.0	0.71
Phenylalanine	Amino Acids	18	42370	74 – 9442	10.8	0.54
Proline	Amino Acids	106	135530	106 – 13550	8.4	0.75
Tryptophane	Amino Acids	27	41630	33 – 4172	9.2	0.68
Tryptophane betaine	Amino Acids	4	12990	27 – 3460	9.0	0.70
Tyrosine	Amino Acids	12	24830	37 – 4703	9.0	0.70
Chenodeoxycholic acid	Bile Acids	25	61130	31 – 3974	8.4	0.75
Cholic acid	Bile Acids	12	29370	16 – 2085	8.9	0.71
Deoxycholic acid	Bile Acids	18	56040	31 – 3974	9.0	0.70
Glycochenodeoxycholic acid	Bile Acids	18	33810	27 – 3470	9.1	0.69
Glycocholic acid	Bile Acids	3	9230	14 – 1830	9.1	0.70
Glycodeoxycholic acid	Bile Acids	3	7560	15 – 1895	9.0	0.70
Glycohyocholic acid	Bile Acids	3	6870	14 – 1830	8.7	0.73
Glycoursodeoxycholic acid	Bile Acids	18	33810	27 – 3470	9.7	0.64
Taurocholic acid	Bile Acids	10	23270	13 – 1652	9.7	0.64
Acetyl-carnitine	Fatty Acyls	1	2710	11 – 1358	8.1	0.77
Butyryl-carnitine	Fatty Acyls	9	11240	15 – 1863	8.7	0.73
Carnitine	Fatty Acyls	9	27870	21 – 2657	8.5	0.75
Decanoyl-carnitine	Fatty Acyls	5	10150	5 – 275	8.7	0.73
Hexanoyl-carnitine	Fatty Acyls	1	2310	4 – 548	8.8	0.72
Lauroyl-carnitine	Fatty Acyls	2	4660	6 – 743	9.1	0.69
Linoleyl-carnitine	Fatty Acyls	2	5380	3 – 335	7.8	0.80
Malonyl-carnitine	Fatty Acyls	19	32940	33 – 4282	9.7	0.64
Octanoyl-carnitine	Fatty Acyls	1	2090	4 – 494	8.8	0.72
Oleoyl-carnitine	Fatty Acyls	33	39940	16 – 2002	8.8	0.72
Palmitoyl-carnitine	Fatty Acyls	3	5330	3 – 355	7.5	0.83
Stearoyl-carnitine	Fatty Acyls	1	2690	3 – 332	7.9	0.79
Tetradecanoyl-carnitine	Fatty Acyls	1	1510	6 – 743	8.6	0.74
Valeryl-carnitine	Fatty Acyls	3	6520	7 – 868	8.5	0.75
C16 Lyso PAF	Glycerophospholipids	8	17190	23 – 2980	8.7	0.73
LysoPC(12:0)	Glycerophospholipids	2	4850	4 – 485	8.9	0.71
LysoPC(14:0)	Glycerophospholipids	2	4560	2 – 304	9.2	0.68
LysoPC(15:0)	Glycerophospholipids	3	6650	4 – 442	8.5	0.74
LysoPC(16:0)	Glycerophospholipids	3	8070	3 – 430	10.2	0.60
LysoPC(17:0)	Glycerophospholipids	4	8040	4 – 541	8.6	0.74
LysoPC(17:1)	Glycerophospholipids	3	6300	4 – 544	8.7	0.73
LysoPC(18:0)	Glycerophospholipids	8	19100	23 – 2980	8.8	0.72
LysoPC(18:1)	Glycerophospholipids	3	16490	7 – 826	9.3	0.68
LysoPE(14:0)	Glycerophospholipids	3	7760	5 – 650	9.0	0.70
LysoPE(16:0)	Glycerophospholipids	9	15040	15 – 1880	9.4	0.67
LysoPE(18:0)	Glycerophospholipids	8	15400	25 – 3240	6.6	0.88
LysoPE(18:1)	Glycerophospholipids	6	17930	7 – 900	8.6	0.73
Biliverdin	Porphyrins and Bilins	2	3780	2 – 244	7.8	0.80
Hypoxanthine	Purines	44	60250	49 – 6260	8.9	0.71
Sphinganine (d18:0)	Sphingolipids	3	9420	4 – 471	10.5	0.57
Sphinganine 1-P	Sphingolipids	4	11300	9 – 1130	8.8	0.72
Sphingosine (d18:1)	Sphingolipids	3	9480	4 – 474	9.0	0.71
Sphingosine 1-P	Sphingolipids	4	11360	9 – 1136	9.0	0.70
Cortisol	Steroids and Steroid Derivatives	10	21300	19 – 2363	8.8	0.72
Cortisone	Steroids and Steroid Derivatives	22	41380	34 – 4302	9.5	0.66

Acronyms: LLOQ, lower limit of quantification; ULOQ, upper limit of quantification; PAF, platelet-activating factor; P, phosphate; LysoPC, lysophosphatidylcholine; LysoPE, lysophosphatidylethanolamine.

Cox proportional hazard models including both the metabolite and its batch as covariates were used to assess potential batch effects on associations with breast cancer incidence. No significant batch effects were observed (mean p-value = 0.71; range: 0.54–0.88), suggesting metabolite batch does not significantly affect the metabolite-survival

Supplementary Table 3. Associations between metabolites and breast cancer incidence from Cox proportional hazard models, NutriNet-Santé case-cohort study, N=2993, 2011-2022.

**Metabolite**	**Sample Description**	**Outliers**	**Model 0 (unadjusted)** *[n=2993, BC events=306, 23,595 person-years]*		**Model 1 (MAIN)** *[n=2993, BC events=306, 23,595 person-years]*			**Model 1–S1 (diet quality-adjusted)** *[n=2993, BC events=306, 23,595 person-years]*		**Model 1–S2 (early diagnosis excluded)** *[n=2907, BC events=220, 23,505 person-years]*		**Model 1–Post (postmenopausal subgroup)** *[n=2105, BC events=261, 16,743 person-years]*		**Model 1–Pre (premonopausal subgroup)** *[n=1165, BC events=45, 9,807 person-years]*	
	**mean (SD)**	**(N)**	**HR (95% CI)**	**p**	**HR (95% CI)**	**p**	**q**	**HR (95% CI)**	**p**	**HR (95% CI)**	**p**	**HR (95% CI)**	**p**	**HR (95% CI)**	**p**
**Glycohyocholic acid**	0.86 (7.2)	13	1.09 (1.02–1.16)	***0.007***	1.10 (1.03–1.17)	***0.005***	***0.242***	1.10 (1.03–1.17)	***0.006***	1.11 (1.03–1.20)	***0.005***	1.10 (1.02–1.18)	***0.017***	1.13 (0.97–1.31)	0.109
**Linoleyl-carnitine**	17.7 (9.6)	0	0.82 (0.69–0.98)	***0.026***	0.81 (0.68–0.96)	***0.018***	0.470	0.83 (0.69–0.99)	***0.039***	0.80 (0.65–0.98)	***0.029***	0.85 (0.70–1.03)	0.088	0.64 (0.39–1.05)	0.080
**Glycoursodeoxycholic acid**	14.0 (44.1)	0	1.03 (1.00–1.06)	***0.041***	1.03 (1.00–1.06)	***0.048***	0.823	1.03 (1.00–1.06)	0.073	1.03 (0.99–1.06)	0.138	1.04 (1.01–1.08)	***0.014***	0.98 (0.90–1.06)	0.575
**Taurocholic acid**	3.0 (26.0)	1	1.04 (1.00–1.08)	***0.047***	1.04 (1.00–1.08)	0.062	0.823	1.04 (1.00–1.09)	***0.036***	1.02 (0.97–1.07)	0.437	1.03 (0.99–1.08)	0.137	1.03 (0.92–1.15)	0.605
**Chenodeoxycholic acid**	19.1 (45.4)	0	0.98 (0.95–1.01)	0.138	0.98 (0.95–1.01)	0.140	0.860	0.98 (0.95–1.01)	0.132	0.97 (0.94–1.00)	***0.049***	0.97 (0.94–1.00)	0.083	1.01 (0.94–1.08)	0.796
**LysoPC(18:1)**	2235.6 (1064.5)	0	0.90 (0.77–1.06)	0.222	0.90 (0.76–1.07)	0.249	0.860	0.90 (0.76–1.07)	0.227	0.95 (0.78–1.16)	0.645	0.96 (0.79–1.16)	0.651	0.60 (0.38–0.96)	***0.032***
Palmitoyl-carnitine	14.1 (7.2)	0	0.89 (0.74–1.08)	0.236	0.85 (0.70–1.02)	0.086	0.860	0.84 (0.69–1.01)	0.069	0.84 (0.68–1.04)	0.115	0.87 (0.70–1.07)	0.181	0.77 (0.49–1.22)	0.268
Butyryl-carnitine	9.3 (7.8)	40	1.08 (0.97–1.21)	0.153	1.09 (0.97–1.21)	0.147	0.860	1.09 (0.98–1.22)	0.128	1.06 (0.93–1.21)	0.355	1.10 (0.97–1.24)	0.141	1.10 (0.82–1.47)	0.524
Sphinganine 1-P	0.28 (0.49)	30	1.04 (0.99–1.10)	0.115	1.04 (0.99–1.09)	0.164	0.860	1.04 (0.99–1.09)	0.161	1.03 (0.97–1.10)	0.276	1.04 (0.98–1.10)	0.168	1.03 (0.88–1.21)	0.726
Biliverdin	22.2 (17.3)	4	0.92 (0.81–1.05)	0.221	0.91 (0.80–1.04)	0.169	0.860	0.92 (0.80–1.04)	0.192	0.90 (0.78–1.05)	0.192	0.93 (0.81–1.07)	0.335	0.82 (0.56–1.20)	0.316
LysoPE(18:0)	281.2 (161.9)	0	0.92 (0.83–1.02)	0.101	0.93 (0.84–1.03)	0.179	0.860	0.94 (0.84–1.04)	0.221	0.95 (0.84–1.08)	0.460	0.94 (0.83–1.05)	0.254	0.92 (0.70–1.20)	0.526
Hypoxanthine	205.1 (164.6)	28	0.96 (0.87–1.05)	0.357	0.94 (0.85–1.04)	0.212	0.860	0.95 (0.86–1.05)	0.281	0.93 (0.83–1.04)	0.197	0.94 (0.84–1.05)	0.266	0.93 (0.71–1.21)	0.583
Cholic acid	14.5 (46.3)	0	0.98 (0.96–1.01)	0.307	0.98 (0.95–1.01)	0.270	0.860	0.98 (0.95–1.01)	0.247	0.98 (0.95–1.01)	0.254	0.99 (0.95–1.02)	0.398	0.96 (0.89–1.04)	0.309
Proline	969.1 (385.6)	0	0.91 (0.72–1.15)	0.426	0.88 (0.69–1.12)	0.288	0.860	0.86 (0.67–1.10)	0.230	0.92 (0.70–1.21)	0.544	0.88 (0.67–1.14)	0.328	0.76 (0.42–1.40)	0.381
Carnitine	231.1 (639)	1	0.91 (0.70–1.19)	0.498	0.86 (0.65–1.13)	0.288	0.860	0.86 (0.65–1.14)	0.301	0.86 (0.63–1.19)	0.374	0.78 (0.57–1.07)	0.122	1.21 (0.64–2.28)	0.553
Glycocholic acid	12.1 (30.4)	0	1.02 (0.99–1.05)	0.277	1.02 (0.98–1.05)	0.292	0.860	1.02 (0.99–1.05)	0.241	1.01 (0.98–1.05)	0.480	1.02 (0.98–1.06)	0.279	0.99 (0.91–1.08)	0.795
Sphingosine (d18:1)	13.7 (7.5)	1	1.09 (0.94–1.27)	0.273	1.08 (0.93–1.26)	0.318	0.860	1.09 (0.93–1.27)	0.295	1.14 (0.95–1.36)	0.163	1.07 (0.91–1.25)	0.437	1.15 (0.67–1.98)	0.616
C16 Lyso PAF	89.7 (105)	0	0.94 (0.84–1.05)	0.293	0.94 (0.84–1.06)	0.336	0.860	0.94 (0.83–1.06)	0.293	0.99 (0.87–1.13)	0.910	0.95 (0.84–1.08)	0.426	0.90 (0.65–1.24)	0.508
Acetyl-carnitine	77.5 (36)	0	0.92 (0.77–1.11)	0.397	0.92 (0.76–1.10)	0.355	0.860	0.92 (0.76–1.11)	0.393	0.86 (0.69–1.07)	0.164	0.92 (0.75–1.13)	0.415	0.94 (0.55–1.59)	0.810
Stearoyl-carnitine	5.4 (3.3)	9	0.95 (0.83–1.08)	0.415	0.94 (0.82–1.07)	0.365	0.860	0.94 (0.82–1.08)	0.367	0.96 (0.82–1.12)	0.578	0.99 (0.86–1.15)	0.918	0.74 (0.50–1.11)	0.146
Tetradecanoyl-carnitine	4.3 (2.8)	27	0.97 (0.87–1.09)	0.595	0.95 (0.84–1.07)	0.367	0.860	0.94 (0.83–1.06)	0.292	0.95 (0.83–1.08)	0.418	0.97 (0.85–1.10)	0.600	0.90 (0.70–1.14)	0.376
Oleoyl-carnitine	24.5 (14.2)	11	0.97 (0.84–1.12)	0.648	0.94 (0.81–1.09)	0.398	0.860	0.94 (0.81–1.10)	0.453	0.93 (0.79–1.10)	0.423	1.00 (0.85–1.18)	0.959	0.76 (0.53–1.08)	0.122
LysoPE(18:1)	245.6 (160.4)	0	0.94 (0.82–1.08)	0.385	0.94 (0.82–1.09)	0.423	0.860	0.94 (0.81–1.09)	0.394	0.96 (0.81–1.13)	0.626	0.97 (0.83–1.13)	0.659	0.74 (0.48–1.13)	0.165
Octanoyl-carnitine	16.4 (15.8)	8	0.97 (0.85–1.10)	0.620	0.95 (0.82–1.09)	0.441	0.860	0.95 (0.83–1.09)	0.472	0.89 (0.76–1.05)	0.179	0.97 (0.83–1.13)	0.687	0.93 (0.69–1.26)	0.635
Valeryl-carnitine	7.9 (5)	11	1.09 (0.94–1.27)	0.251	1.06 (0.91–1.23)	0.443	0.860	1.05 (0.90–1.23)	0.497	1.08 (0.92–1.28)	0.344	1.06 (0.91–1.24)	0.472	1.07 (0.66–1.76)	0.774
Lauroyl-carnitine	10.0 (13.0)	34	0.98 (0.91–1.06)	0.673	0.97 (0.89–1.05)	0.462	0.860	0.97 (0.90–1.06)	0.496	0.95 (0.87–1.04)	0.282	0.99 (0.91–1.08)	0.832	0.93 (0.78–1.11)	0.431
Decanoyl-carnitine	44.7 (39.9)	3	0.97 (0.86–1.09)	0.612	0.96 (0.85–1.08)	0.472	0.860	0.96 (0.85–1.08)	0.497	0.90 (0.78–1.03)	0.130	0.98 (0.85–1.12)	0.751	0.90 (0.69–1.17)	0.429
LysoPC(18:0)	3378 (1334.7)	2	0.91 (0.72–1.16)	0.452	0.91 (0.71–1.17)	0.472	0.860	0.91 (0.71–1.17)	0.476	0.97 (0.73–1.29)	0.831	0.96 (0.74–1.26)	0.787	0.64 (0.33–1.26)	0.199
LysoPC(16:0)	12926.8 (4004)	19	0.91 (0.68–1.22)	0.528	0.90 (0.66–1.21)	0.482	0.860	0.89 (0.66–1.21)	0.464	0.91 (0.64–1.29)	0.585	0.95 (0.69–1.33)	0.780	0.52 (0.21–1.26)	0.148
Glycodeoxycholic acid	25.4 (47)	0	1.01 (0.98–1.05)	0.452	1.01 (0.98–1.05)	0.493	0.860	1.01 (0.98–1.05)	0.476	1.02 (0.98–1.07)	0.280	1.01 (0.97–1.05)	0.576	1.00 (0.92–1.10)	0.920
LysoPC(17:1)	55.4 (47.1)	4	0.95 (0.83–1.08)	0.425	0.96 (0.84–1.09)	0.511	0.860	0.95 (0.83–1.09)	0.430	0.93 (0.80–1.09)	0.381	0.97 (0.84–1.12)	0.657	0.90 (0.61–1.33)	0.609
Cortisone	6.6 (6.0)	0	0.99 (0.94–1.03)	0.520	0.99 (0.94–1.03)	0.519	0.860	0.98 (0.94–1.03)	0.505	0.97 (0.93–1.02)	0.308	0.99 (0.95–1.04)	0.739	0.90 (0.80–1.01)	0.070
Cortisol	35.5 (30.2)	62	0.97 (0.88–1.07)	0.523	0.97 (0.88–1.07)	0.577	0.903	0.97 (0.88–1.07)	0.570	0.94 (0.85–1.04)	0.226	0.95 (0.86–1.06)	0.368	1.02 (0.78–1.33)	0.892
LysoPC(17:0)	460.6 (221)	1	0.93 (0.77–1.11)	0.426	0.95 (0.78–1.15)	0.582	0.903	0.94 (0.77–1.14)	0.542	0.96 (0.77–1.20)	0.741	1.01 (0.82–1.24)	0.943	0.64 (0.37–1.11)	0.110
LysoPC(14:0)	199.1 (101.8)	1	0.99 (0.83–1.19)	0.926	0.95 (0.79–1.14)	0.597	0.903	0.93 (0.77–1.12)	0.450	1.05 (0.85–1.29)	0.659	0.98 (0.80–1.19)	0.826	0.77 (0.47–1.26)	0.296
Hexanoylcarnitine	4.3 (4.3)	39	1.00 (0.90–1.12)	0.966	0.97 (0.87–1.09)	0.643	0.947	0.97 (0.86–1.09)	0.625	0.96 (0.84–1.09)	0.550	0.99 (0.86–1.13)	0.837	0.96 (0.76–1.21)	0.724
LysoPC(15:0)	142.3 (67)	13	0.96 (0.79–1.16)	0.657	0.96 (0.79–1.17)	0.687	0.966	0.94 (0.76–1.15)	0.531	0.99 (0.79–1.24)	0.905	1.00 (0.80–1.23)	0.964	0.72 (0.39–1.34)	0.302

Tryptophane betaine	40.3 (64.3)	0	0.99 (0.96–1.03)	0.732	0.99 (0.96–1.03)	0.693	0.966	1.00 (0.96–1.03)	0.953	1.01 (0.97–1.05)	0.717	0.98 (0.95–1.02)	0.404	1.04 (0.93–1.17)	0.464
Tyrosine	311.2 (98.8)	0	0.99 (0.74–1.33)	0.948	0.95 (0.70–1.27)	0.713	0.969	0.94 (0.70–1.27)	0.696	1.12 (0.82–1.54)	0.482	0.93 (0.68–1.28)	0.648	0.95 (0.44–2.06)	0.905
Methionine	118.9 (38.2)	1	0.95 (0.73–1.24)	0.707	0.95 (0.73–1.25)	0.733	0.972	0.95 (0.72–1.25)	0.700	1.13 (0.83–1.55)	0.428	0.92 (0.69–1.23)	0.569	1.05 (0.54–2.02)	0.888
Leucine	2235.4 (548)	2	1.00 (0.69–1.45)	0.993	0.95 (0.66–1.39)	0.803	0.979	0.94 (0.64–1.37)	0.739	1.02 (0.66–1.56)	0.931	0.97 (0.66–1.44)	0.880	0.84 (0.27–2.60)	0.759
Sphingosine 1-P	2.0 (3.6)	25	1.00 (0.95–1.06)	0.906	0.99 (0.94–1.05)	0.809	0.979	0.99 (0.93–1.05)	0.770	1.01 (0.94–1.08)	0.751	1.00 (0.93–1.06)	0.938	0.96 (0.84–1.11)	0.597
Arginine	112.5 (67.1)	12	0.99 (0.87–1.14)	0.927	1.02 (0.88–1.17)	0.826	0.979	1.01 (0.87–1.17)	0.890	1.00 (0.84–1.18)	0.963	0.98 (0.84–1.14)	0.766	1.23 (0.84–1.81)	0.292
Sphinganine (d18:0)	3.7 (4.4)	0	0.99 (0.94–1.05)	0.724	0.99 (0.94–1.05)	0.827	0.979	1.00 (0.94–1.05)	0.887	1.02 (0.95–1.09)	0.618	1.00 (0.94–1.06)	0.936	0.96 (0.79–1.17)	0.688
Phenylalanine	1453.9 (688.8)	0	1.00 (0.83–1.20)	0.961	0.98 (0.81–1.18)	0.850	0.979	0.99 (0.82–1.19)	0.882	0.99 (0.80–1.24)	0.954	1.01 (0.83–1.24)	0.907	0.84 (0.50–1.40)	0.503
Kynurenine	32.1 (15.2)	5	1.03 (0.87–1.23)	0.710	1.02 (0.85–1.22)	0.851	0.979	1.02 (0.85–1.22)	0.850	1.04 (0.84–1.28)	0.731	0.97 (0.80–1.19)	0.797	1.33 (0.80–2.23)	0.277
Malonylcarnitine	0.58 (1.5)	0	1.00 (0.95–1.06)	0.880	1.00 (0.94–1.05)	0.885	0.979	0.99 (0.94–1.05)	0.829	0.98 (0.92–1.05)	0.628	1.01 (0.95–1.07)	0.738	0.93 (0.78–1.11)	0.407
Tryptophane	2031.4 (495.7)	7	0.99 (0.69–1.42)	0.967	0.98 (0.68–1.41)	0.918	0.979	0.96 (0.66–1.37)	0.806	1.15 (0.76–1.74)	0.511	0.94 (0.65–1.38)	0.760	1.24 (0.38–4.04)	0.716
Glycochenodeoxycholic acid	57.1 (80.8)	1	1.00 (0.96–1.04)	0.905	1.00 (0.96–1.04)	0.924	0.979	1.01 (0.96–1.05)	0.795	0.99 (0.95–1.04)	0.765	1.00 (0.96–1.05)	0.927	1.00 (0.90–1.10)	0.926
Deoxycholic acid	22.8 (42.5)	0	1.00 (0.97–1.03)	0.838	1.00 (0.97–1.03)	0.929	0.979	1.00 (0.97–1.03)	0.930	1.00 (0.97–1.04)	0.997	1.00 (0.97–1.04)	0.787	0.97 (0.90–1.05)	0.514
LysoPC(12:0)	1.5 (2.5)	0	1.00 (0.96–1.05)	0.844	1.00 (0.96–1.04)	0.942	0.979	0.99 (0.95–1.04)	0.804	1.01 (0.96–1.06)	0.726	1.01 (0.97–1.06)	0.590	0.92 (0.83–1.02)	0.127
LysoPE(14:0)	0.88 (1.5)	0	1.00 (0.95–1.05)	0.995	1.00 (0.95–1.05)	0.965	0.983	1.00 (0.95–1.05)	0.863	1.01 (0.95–1.07)	0.706	1.00 (0.95–1.06)	0.999	0.96 (0.84–1.09)	0.497
LysoPE(16:0)	292 (146.4)	6	0.98 (0.82–1.18)	0.867	1.00 (0.83–1.21)	0.986	0.986	1.00 (0.83–1.21)	0.992	1.04 (0.84–1.29)	0.711	1.02 (0.83–1.25)	0.838	0.80 (0.45–1.42)	0.441

Abbreviations: BC, breast cancer; CI, confidence interval; HR, hazard ratio.
Cox proportional hazard models were used to investigate the independent associations between metabolites and breast cancer risk. Participant age was used as the timescale to model time-to-event with ISSP weightings. Metabolite values were $\log_2$-transformed; hazard ratios reflect the change in risk per doubling of metabolite concentration. P-values were adjusted (q vals) for false-discovery rate using the Benjamini-Hochberg procedure.
Model 0 was unadjusted.
Model 1 was the main model, adjusted for waist circumference (continuous), height (continuous), education level (< high-school degree, ≤ 3 years of higher education, >3 years of higher education), family history of cancer (all types, immediate family; yes/no), prevalent health condition at baseline or diagnosed within one year follow-up (includes type II diabetes, hypertension, hypercholesterolemia), smoking status (never, former, current) and smoking pack-years (continuous), alcohol intake (g/day), physical activity (IPAQ categories: low, medium, high), age at menarche (<12, 12–15, >15 years), number of biological children (continuous), age at first birth (no children, <20 year, 20–24, 25–34, ≥35 years), contraceptive use (ever; yes/no), menopausal status at baseline (pre/postmenopausal), and an interaction term between menopausal status and waist circumference.
Model 1–S1 was model 1, additionally number of R24Hs (continuous), time from last R24H to baseline (continuous), total energy intake (kcal/day, excluding alcohol), and dietary patterns derived by principal component analysis.
Model 1–S2 was model 1 excluding cases diagnosed within the first two years of follow-up (n=86) as a sensitivity analysis.
Model 1–Post and Model 1–Pre were adjusted as in model 1 except excluding menopause status and interaction term, and recategorizing age of first birth (no children, <30, ≥30 years) to account for smaller sample sizes. Model 1–Post additionally adjusted for age at menopause and menopause hormone therapy use.

Supplementary Table 4. Cox proportional hazard models of metabolite patterns based 11 principal components to breast cancer incidence, NutriNet-Santé case-cohort study, N=2993, 2011-2022.

**Principal Component**	**Model 0 (unadjusted)** *[n=2993, BC events=306, 23,595 person-years]*		**Model 1 (MAIN)** *[n=2993, BC events=306, 23,595 person-years]*		**Model 1–S1 (diet quality-adjusted)** *[n=2993, BC events=306, 23,595 person-years]*		**Model 1–S2 (early diagnosis excluded)** *[n=2907, BC events=220, 23,505 person-years]*		**Model 1–Post (postmenopausal subgroup)** *[n=2105, BC events=261, 16,743 person-years]*		**Model 1–Pre (premonopausal subgroup)** *[n=1165, BC events=45, 9,807 person-years]*	
	**HR (95% CI)**	**p**	**HR (95% CI)**	**p**	**HR (95% CI)**	**p**	**HR (95% CI)**	**p**	**HR (95% CI)**	**p**	**HR (95% CI)**	**p**
**PC 04**	1.14 (1.00–1.30)	***0.047***	1.14 (1.00–1.30)	***0.049***	1.15 (1.01–1.32)	***0.039***	1.13 (0.97–1.32)	0.108	1.15 (0.99–1.33)	0.062	1.05 (0.76–1.44)	0.772
**PC 09**	0.91 (0.81–1.02)	0.121	0.90 (0.80–1.02)	0.092	0.90 (0.80–1.01)	0.076	0.86 (0.75–0.99)	***0.036***	0.91 (0.80–1.03)	0.146	0.86 (0.63–1.18)	0.350
PC 02	0.94 (0.83–1.08)	0.385	0.92 (0.80–1.05)	0.207	0.92 (0.80–1.06)	0.226	0.85 (0.72–1.00)	0.050	0.93 (0.79–1.09)	0.379	0.92 (0.69–1.22)	0.550
PC 07	0.92 (0.81–1.04)	0.166	0.93 (0.81–1.05)	0.247	0.94 (0.83–1.08)	0.394	0.97 (0.83–1.14)	0.725	0.91 (0.79–1.05)	0.214	1.03 (0.74–1.42)	0.881
PC 01	0.94 (0.83–1.06)	0.295	0.94 (0.84–1.07)	0.350	0.94 (0.83–1.06)	0.288	1.00 (0.86–1.15)	0.966	0.97 (0.85–1.11)	0.675	0.75 (0.54–1.04)	0.084
PC 03	1.05 (0.93–1.19)	0.399	1.04 (0.92–1.17)	0.554	1.04 (0.92–1.17)	0.581	1.09 (0.95–1.24)	0.207	1.02 (0.90–1.16)	0.740	1.12 (0.79–1.59)	0.519
PC 10	1.00 (0.91–1.11)	0.930	1.02 (0.92–1.14)	0.683	1.02 (0.92–1.14)	0.693	1.00 (0.88–1.13)	0.949	1.00 (0.89–1.11)	0.973	1.16 (0.84–1.60)	0.358
PC 06	1.01 (0.89–1.16)	0.844	1.02 (0.89–1.17)	0.757	1.02 (0.89–1.17)	0.764	1.03 (0.88–1.20)	0.711	1.01 (0.87–1.16)	0.930	1.00 (0.69–1.45)	0.991
PC 05	1.03 (0.92–1.16)	0.584	1.02 (0.91–1.14)	0.761	1.01 (0.90–1.14)	0.815	1.02 (0.89–1.17)	0.742	1.00 (0.88–1.13)	0.950	1.16 (0.83–1.62)	0.375
PC 11	1.01 (0.90–1.13)	0.840	1.01 (0.90–1.13)	0.893	1.01 (0.90–1.13)	0.907	1.02 (0.89–1.18)	0.725	1.01 (0.89–1.15)	0.881	1.00 (0.73–1.35)	0.975
PC 08	0.98 (0.86–1.11)	0.719	0.99 (0.87–1.13)	0.918	1.00 (0.88–1.14)	0.999	1.02 (0.87–1.19)	0.824	0.97 (0.85–1.12)	0.679	1.15 (0.81–1.64)	0.437

Abbreviations: BC, breast cancer; CI, confidence interval; HR, hazard ratio.

Principal component analysis with varimax rotation on 11 factors was used to identify metabolite patterns (Supplementary Figure 2).

Cox proportional hazard models were used to investigate the independent associations between metabolite patterns and breast cancer risk. Participant age was used as the timescale to model time-to-event with ISSP weightings.

Model 0 was unadjusted.

Model 1 was the main model, adjusted for waist circumference (continuous), height (continuous), education level (< high-school degree, ≤ 3 years of higher education, >3 years of higher education), family history of cancer (all types, immediate family; yes/no), prevalent health condition at baseline or diagnosed within one year follow-up (includes type II diabetes, hypertension, hypercholesterolemia), smoking status (never, former, current) and smoking pack-years (continuous), alcohol intake (g/day), physical activity (IPAQ categories: low, medium, high), age at menarche (<12, 12–15, >15 years), number of biological children (continuous), age at first birth (no children, <20 year, 20–24, 25–34, ≥35 years), contraceptive use (ever; yes/no), menopausal status at baseline (pre/postmenopausal), and an interaction term between menopausal status and waist circumference.

Model 1–S1 was model 1, additionally number of R24Hs (continuous), time from last R24H to baseline (continuous), total energy intake (kcal/day, excluding alcohol), and dietary patterns derived by principal component analysis.

Model 1–S2 was model 1 excluding cases diagnosed within the first two years of follow-up (n=86) as a sensitivity analysis.

Model 1–Post and Model 1–Pre were adjusted as in model 1 except excluding menopause status and interaction term, and recategorizing age of first birth (no children, <30, ≥30 years) to account for smaller sample sizes. Model 1–Post additionally adjusted for age at menopause and menopause hormone therapy use.

Supplementary Table 5. Diet–metabolite associations selected from ElasticNet regression and subsequently jointly fit as predictors in linear models per metabolite to estimate associations, NutriNet-Santé case-cohort study, N=2993, 2011-2022.

Main Model (Model 1–S1, excluding PC scores, with age) *[n=2993, BC events=306, 23,595 person-years]*						Sensitivity Model (Model 1–S3, excluding extreme energy intake) *[n=2963, BC events=301, 23,303 person-years]*					
**ElasticNet**			**Linear Models ElasticNet-selected predictors**			**ElasticNet**			**Linear Models ElasticNet-selected predictors**		
**Metabolite**	**Selected Dietary Variables**	**Coefficient**	**β (95% CI)**	**2^β (% change)**	**p value**	**Metabolite**	**Selected Dietary Variables**	**Coefficient**	**β (95% CI)**	**2^β (% change)**	**p value**
**Glycohyocholic acid**	(n=12)					**Glycohyocholic acid**	(n=12)				
α=0.1, λ=0.281	**Vitamin K**	**-0.033**	**-0.13 (-0.20, -0.05)**	**-8.39**	**0.001**	α=0.1, λ=0.281	**Vitamin K**	**-0.033**	**-0.13 (-0.20, -0.05)**	**-8.37**	**0.001**
RMSE = 1.83 (SD 0.072)	**Processed meats**	**-0.054**	**-0.09 (-0.16, -0.02)**	**-5.94**	**0.017**	RMSE = 1.83 (SD 0.08)	**Processed meats**	**-0.051**	**-0.09 (-0.16, -0.01)**	**-5.72**	**0.022**
R2 = 0.008 (SD 0.009)	**Plant lipids**	**0.033**	**0.11 (0.01, 0.20)**	**7.57**	**0.028**	R2 = 0.010 (SD 0.012)	**Plant lipids**	**0.036**	**0.11 (0.01, 0.20)**	**7.83**	**0.023**
	**Refined grains**	**0.013**	**0.09 (0.00, 0.18)**	**6.50**	**0.046**		Refined grains	0.012	0.09 (0.00, 0.18)	6.40	0.050
	Vegetables	0.000	0.00 (0.00, 0.00)	0.05	0.061		Vegetables	0.000	0.00 (0.00, 0.00)	0.05	0.065
	Legumes	0.028	0.07 (-0.01, 0.15)	4.87	0.084		Legumes	0.031	0.07 (-0.01, 0.15)	5.19	0.067
	Potatoes and tubers	-0.008	-0.05 (-0.12, 0.02)	-3.34	0.162		Iodine	0.013	0.05 (-0.02, 0.12)	3.56	0.143
	Iodine	0.010	0.05 (-0.02, 0.12)	3.39	0.163		Potatoes and tubers	-0.007	-0.05 (-0.12, 0.02)	-3.25	0.176
	Mn	0.002	0.05 (-0.05, 0.15)	3.35	0.353		Mn	0.001	0.05 (-0.06, 0.15)	3.21	0.374
	Soluble fiber	0.024	0.05 (-0.08, 0.18)	3.65	0.429		Soluble fiber	0.026	0.06 (-0.07, 0.18)	3.90	0.400
	Cakes and biscuits	-0.010	-0.03 (-0.11, 0.05)	-2.10	0.465		Cakes and biscuits	-0.009	-0.03 (-0.11, 0.05)	-2.02	0.485
	Vegetable protein	0.019	-0.04 (-0.20, 0.12)	-2.64	0.642		Vegetable protein	0.020	-0.04 (-0.20, 0.12)	-2.68	0.638
**Linoleyl-carnitine**	(n=30)					**Linoleyl-carnitine**	(n=30)				
α=0.1, λ=0.061	**Breakfast cereals**	**0.027**	**0.05 (0.02, 0.08)**	**3.63**	**0.0004**	α=0.1, λ=0.071	**Breakfast cereals**	**0.024**	**0.04 (0.02, 0.07)**	**3.02**	**0.002**
RMSE = 0.68 (SD 0.02)	**Milk and soft cheeses**	**-0.034**	**-0.05 (-0.08, -0.02)**	**-3.23**	**0.002**	RMSE = 0.68 (SD 0.023)	**Potatoes and tubers**	**0.028**	**0.04 (0.01, 0.07)**	**2.84**	**0.004**
R2 = 0.057 (SD 0.022)	**Potatoes and tubers**	**0.029**	**0.04 (0.01, 0.07)**	**2.82**	**0.003**	R2 = 0.057 (SD 0.023)	**Yogurt**	**-0.028**	**-0.04 (-0.07, -0.01)**	**-2.80**	**0.005**
	**Yogurt**	**-0.028**	**-0.04 (-0.07, -0.01)**	**-2.83**	**0.004**		**Milk and soft cheeses**	**-0.032**	**-0.04 (-0.07, -0.01)**	**-2.91**	**0.006**
	**Cakes and biscuits**	**0.024**	**0.05 (0.01, 0.09)**	**3.67**	**0.007**		**Cakes and biscuits**	**0.018**	**0.05 (0.01, 0.09)**	**3.39**	**0.020**
	**Vitamin E**	**0.042**	**0.05 (0.01, 0.10)**	**3.62**	**0.028**		**Vitamin E**	**0.041**	**0.06 (0.01, 0.11)**	**4.12**	**0.025**
	Vitamin B3	-0.004	-0.03 (-0.07, 0.01)	-2.27	0.104		Legumes	0.016	0.03 (0.00, 0.05)	1.77	0.090
	Fats and oils	0.012	0.03 (-0.01, 0.06)	1.95	0.113		Fats and oils	0.011	0.03 (-0.01, 0.06)	2.00	0.101
	Legumes	0.018	0.02 (-0.01, 0.05)	1.57	0.131		Oilseeds	0.007	0.03 (-0.01, 0.06)	1.85	0.156
	SFAs	-0.017	-0.08 (-0.18, 0.03)	-5.28	0.145		Red meat and offal	-0.015	-0.02 (-0.05, 0.01)	-1.47	0.180
	Alpha-linolenic acid	0.014	0.03 (-0.01, 0.06)	1.79	0.162		Refined grains	0.011	0.03 (-0.01, 0.07)	1.83	0.187
	Red meat and offal	-0.017	-0.02 (-0.05, 0.01)	-1.51	0.173		Alpha-linolenic acid	0.011	0.02 (-0.01, 0.06)	1.67	0.200
	Oilseeds	0.009	0.02 (-0.01, 0.06)	1.56	0.200		Vitamin B12	-0.007	-0.02 (-0.05, 0.01)	-1.16	0.253
	Sodium	0.011	0.02 (-0.01, 0.06)	1.71	0.217		Sodium	0.008	0.02 (-0.02, 0.06)	1.56	0.263
	Refined grains	0.015	0.02 (-0.02, 0.06)	1.55	0.249		Unsweetened soft drinks	0.007	0.02 (-0.01, 0.04)	1.07	0.263
	Unsweetened soft drinks	0.007	0.02 (-0.01, 0.04)	1.07	0.258		Vitamin C	-0.007	-0.02 (-0.05, 0.01)	-1.13	0.266
	Vitamin B12	-0.008	-0.01 (-0.04, 0.01)	-1.01	0.305		Fruits	-0.002	-0.02 (-0.06, 0.02)	-1.37	0.296
	Sugar and confectionary	-0.013	-0.02 (-0.05, 0.02)	-1.17	0.318		Sugar and confectionary	-0.013	-0.02 (-0.05, 0.02)	-1.26	0.305
	Vitamin C	-0.008	-0.01 (-0.04, 0.01)	-1.00	0.325		Animal protein	-0.028	-0.03 (-0.08, 0.03)	-1.90	0.313
	Fruits	-0.002	-0.02 (-0.05, 0.02)	-1.13	0.353		SFAs	-0.011	-0.06 (-0.18, 0.07)	-3.96	0.360
	Plant protein	0.023	0.02 (-0.04, 0.09)	1.59	0.492		Eicosapentaenoic acid	0.000	0.01 (-0.02, 0.04)	0.72	0.494
	Eicosapentaenoic acid	0.001	0.01 (-0.02, 0.04)	0.70	0.504		Sweetened soft drinks	-0.003	-0.01 (-0.04, 0.02)	-0.69	0.531
	Sweetened soft drinks	-0.005	-0.01 (-0.04, 0.02)	-0.70	0.505		Vitamin K	0.005	0.01 (-0.02, 0.04)	0.63	0.559
	Vegetables	0.000	0.00 (0.00, 0.00)	0.01	0.537		Vegetables	0.000	0.00 (0.00, 0.00)	0.01	0.650
	Vitamin K	0.005	0.01 (-0.02, 0.04)	0.50	0.640		Plant lipids	0.001	-0.02 (-0.11, 0.08)	-1.26	0.705
	Beta-carotene	0.004	0.01 (-0.03, 0.04)	0.49	0.681		Beta-carotene	0.004	0.01 (-0.03, 0.04)	0.44	0.712
	Animal protein	-0.025	-0.01 (-0.06, 0.05)	-0.41	0.840		Cholesterol	0.000	-0.01 (-0.05, 0.03)	-0.47	0.744
	Cheeses	0.000	0.00 (-0.04, 0.03)	-0.23	0.850		Linoleic acid	0.014	0.01 (-0.05, 0.06)	0.45	0.807
	Animal lipids	-0.017	0.00 (-0.08, 0.09)	0.21	0.945		Plant protein	0.025	0.01 (-0.06, 0.08)	0.59	0.815
	Linoleic acid	0.013	0.00 (-0.05, 0.05)	0.04	0.980		Animal lipids	-0.015	-0.01 (-0.15, 0.13)	-0.56	0.911
**Glycoursodeoxycholic acid**	(n=22)					**Glycoursodeoxycholic acid**	(n=23)				
α=0.2, λ=0.191	**Oilseeds**	**-0.255**	**-0.36 (-0.53, -0.18)**	**-21.96**	**6.31E-05**	α=0.2, λ=0.201	**Oilseeds**	**-0.250**	**-0.36 (-0.53, -0.18)**	**-21.98**	**0.000**
RMSE = 3.97 (SD 0.06)	**Vitamin E**	**0.168**	**0.42 (0.20, 0.64)**	**33.78**	**0.0001**	RMSE = 3.97 (SD 0.06)	**Vitamin E**	**0.156**	**0.42 (0.21, 0.64)**	**34.17**	**0.000**
R2 = 0.016 (0.013)	**Docosapentaenoic acid**	**0.035**	**0.20 (0.02, 0.39)**	**15.06**	**0.033**	R2 = 0.016 (SD 0.011)	**Complex Carbs**	**0.130**	**0.28 (0.02, 0.53)**	**21.04**	**0.033**

	**Complex Carbs**	**0.134**	**0.27 (0.02, 0.52)**	**20.54**	**0.034**		**Docosapentaenoic acid**	**0.032**	**0.20 (0.02, 0.39)**	**15.13**	**0.033**
	**Docosahexaenoic acid**	**-0.063**	**-0.21 (-0.41, -0.01)**	**-13.65**	**0.037**		**Docosahexaenoic acid**	**-0.055**	**-0.21 (-0.41, -0.01)**	**-13.36**	**0.043**
	Eggs	-0.075	-0.15 (-0.33, 0.02)	-10.12	0.088		Eggs	-0.077	-0.16 (-0.33, 0.02)	-10.32	0.083
	Mg	-0.151	-0.18 (-0.38, 0.03)	-11.54	0.090		Mg	-0.140	-0.16 (-0.37, 0.06)	-10.20	0.156
	Potatoes and tubers	0.059	0.12 (-0.04, 0.28)	8.79	0.131		Legumes	-0.044	-0.11 (-0.25, 0.04)	-7.03	0.169
	Legumes	-0.047	-0.11 (-0.25, 0.04)	-7.04	0.166		Cheeses	0.014	0.12 (-0.05, 0.29)	8.67	0.175
	Cu	-0.043	-0.11 (-0.28, 0.06)	-7.44	0.192		Potatoes and tubers	0.039	0.10 (-0.05, 0.26)	7.45	0.199
	Cheeses	0.013	0.11 (-0.06, 0.28)	7.96	0.207		Fruits	-0.073	-0.10 (-0.28, 0.07)	-6.86	0.247
	Fruits	-0.073	-0.1 (-0.28, 0.07)	-6.92	0.240		Breakfast cereals	0.031	0.10 (-0.07, 0.26)	7.00	0.248
	Breakfast cereals	0.026	0.09 (-0.08, 0.26)	6.44	0.285		Cakes and biscuits	0.049	0.10 (-0.08, 0.29)	7.36	0.275
	Cakes and biscuits	0.050	0.10 (-0.08, 0.28)	7.07	0.288		Cu	-0.027	-0.09 (-0.26, 0.08)	-6.34	0.276
	Processed meats	-0.020	-0.08 (-0.25, 0.08)	-5.54	0.330		Arachidonic acid	-0.165	-0.1 (-0.33, 0.13)	-6.66	0.393
	Vitamin B5	-0.066	-0.15 (-0.49, 0.19)	-9.87	0.389		Processed meats	-0.007	-0.07 (-0.23, 0.10)	-4.61	0.422
	Arachidonic acid	-0.163	-0.1 (-0.32, 0.13)	-6.46	0.405		Vitamin B5	-0.057	-0.14 (-0.48, 0.21)	-9.08	0.434
	Red meat and offal	-0.033	-0.08 (-0.28, 0.11)	-5.65	0.406		Yogurt	-0.038	-0.06 (-0.22, 0.10)	-3.94	0.484
	Yogurt	-0.035	-0.05 (-0.22, 0.11)	-3.67	0.513		Red meat and offal	-0.018	-0.07 (-0.27, 0.13)	-4.65	0.499
	Vitamin C	-0.005	-0.04 (-0.2, 0.12)	-2.86	0.601		Vitamin C	-0.005	-0.04 (-0.20, 0.11)	-2.97	0.587
	Vitamin B2	-0.060	-0.02 (-0.34, 0.31)	-1.04	0.927		Unsweetened soft drinks	-0.007	-0.04 (-0.20, 0.12)	-2.90	0.601
	FeH	-0.002	-0.01 (-0.19, 0.18)	-0.58	0.930		Vitamin B2	-0.079	-0.04 (-0.36, 0.29)	-2.53	0.825
							FeH	-0.002	-0.01 (-0.20, 0.18)	-0.69	0.916
**Taurocholic acid**	(n=4)					**Taurocholic acid**	(n=4)				
α=1, λ=0.061	**Soups and broths**	**0.084**	**0.17 (0.07, 0.27)**	**12.53**	**0.001**	α=0.2, λ=0.291	**Soups and broths**	**0.073**	**0.16 (0.06, 0.27)**	**12.04**	**0.002**
RMSE = 2.75 (SD 0.08)	**Breakfast cereals**	**0.044**	**0.12 (0.02, 0.22)**	**8.87**	**0.017**	RMSE = 2.75 (SD 0.09)	**Breakfast cereals**	**0.040**	**0.12 (0.02, 0.22)**	**8.67**	**0.020**
R2 = 0.005 (SD 0.007)	Red meat and offal	-0.016	-0.08 (-0.18, 0.03)	-5.33	0.141	R2 = 0.006 (SD 0.007)	Red meat and offal	-0.016	-0.08 (-0.18, 0.03)	-5.18	0.156
	Arachidonic acid	0.000	-0.07 (-0.18, 0.04)	-4.60	0.217		Arachidonic acid	-0.004	-0.07 (-0.18, 0.04)	-4.77	0.203
**Chenodeoxycholic acid**	(n=10)					**Chenodeoxycholic acid**	(n=9)				
α=0.4, λ=0.161	**Beta-carotene**	**0.145**	**0.26 (0.10, 0.42)**	**20.03**	**0.001**	α=0.6, λ=0.111	**Beta-carotene**	**0.141**	**0.27 (0.11–0.43)**	**20.68**	**0.001**
RMSE = 4.22 (SD 0.05)	**Oilseeds**	**-0.182**	**-0.28 (-0.45, -0.11)**	**-17.50**	**0.001**	RMSE = 4.22 (SD 0.05)	**Oilseeds**	**-0.191**	**-0.27 (-0.44–-0.1)**	**-17.08**	**0.002**
R2 = 0.007 (SD 0.008)	**Milk and soft cheeses**	**-0.090**	**-0.19 (-0.35, -0.03)**	**-12.43**	**0.018**	R2 = 0.006 (SD 0.006)	**Milk and soft cheeses**	**-0.092**	**-0.18 (-0.34–-0.02)**	**-11.86**	**0.023**
	Cu	-0.082	-0.17 (-0.34, 0.00)	-10.85	0.056		**Cu**	**-0.097**	**-0.18 (-0.35–-0.01)**	**-11.62**	**0.041**
	FeH	-0.059	-0.15 (-0.30, 0.01)	-9.67	0.068		Potatoes and tubers	0.042	0.14 (-0.02–0.3)	10.17	0.085
	Potatoes and tubers	0.041	0.13 (-0.03, 0.29)	9.44	0.107		FeH	-0.054	-0.14 (-0.3–0.02)	-9.08	0.088
	Cakes and biscuits	0.049	0.14 (-0.05, 0.33)	10.06	0.157		Cakes and biscuits	0.054	0.16 (-0.03–0.35)	12.10	0.090
	Sugar and confectionary	-0.006	-0.12 (-0.29, 0.06)	-7.71	0.190		Refined grains	0.034	0.13 (-0.06–0.32)	9.50	0.183
	Refined grains	0.056	0.13 (-0.07, 0.32)	9.10	0.205		Whole grains	-0.007	-0.06 (-0.23–0.12)	-3.85	0.527
	Whole grains	-0.014	-0.08 (-0.25, 0.10)	-5.29	0.384						
**LysoPC(18:1)**	(n=17)					**LysoPC(18:1)**	(n=0)				
α=0.1, λ=0.081	**Linoleic acid**	**-0.009**	**-0.06 (-0.11, -0.01)**	**-4.19**	**0.011**	α=0.1, λ=1.091					
RMSE = 0.73 (SD 0.02)	**Potatoes and tubers**	**0.014**	**0.03 (0.01, 0.06)**	**2.28**	**0.020**	RMSE = 0.73 (SD 0.024)					
R2 = 0.032 (SD 0.019)	**Cakes and biscuits**	**0.017**	**0.04 (0.00, 0.07)**	**2.61**	**0.024**	R2 = 0.031 (SD 0.018)					
	**Caffeine**	**0.018**	**0.03 (0.00, 0.06)**	**2.36**	**0.028**						
	Vitamin C	-0.016	-0.03 (-0.06, 0.00)	-1.90	0.055						
	Fe	0.006	0.04 (0.00, 0.08)	2.75	0.061						
	Vitamin B2	-0.007	-0.03 (-0.07, 0.02)	-1.87	0.214						
	Unsweetened soft drinks	0.009	0.02 (-0.01, 0.05)	1.29	0.214						
	Vegetable lipids	0.006	0.05 (-0.03, 0.12)	3.23	0.248						
	MUFAs	0.008	0.03 (-0.04, 0.11)	2.38	0.370						
	Arachidonic acid	-0.007	-0.01 (-0.05, 0.02)	-1.00	0.412						
	P	0.000	-0.02 (-0.07, 0.03)	-1.38	0.455						
	Processed meats	-0.004	-0.01 (-0.04, 0.02)	-0.61	0.567						
	Poultry	-0.001	-0.01 (-0.04, 0.02)	-0.52	0.613						
	Sweetened soft drinks	0.000	0.00 (-0.03, 0.03)	-0.29	0.780						
	Milk and soft cheeses	-0.002	0.00 (-0.03, 0.04)	0.27	0.812						
	Animal protein	-0.011	0.00 (-0.05, 0.05)	0.23	0.894						
**Principal Component 04**	(n=22)					**Principal Component 04**	(n=24)				
α=0.1, λ=0.131	**Breakfast cereals**	**0.024**	**0.07 (0.03, 0.12)**	**5.06**	**0.002**	α=0.1, λ=0.131	**Vitamin K**	**-0.023**	**-0.07 (-0.12, -0.03)**	**-4.88**	**0.001**

RMSE = 0.99 (SD 0.03)	**Vitamin K**	**-0.022**	**-0.07 (-0.11, -0.02)**	**-4.46**	**0.002**	RMSE = 0.99 (SD 0.03)	**Breakfast cereals**	**0.023**	**0.08 (0.03, 0.12)**	**5.38**	**0.001**
R2 = 0.023 (SD 0.014)	**Vegetables**	**0.000**	**0.00 (0.00, 0.00)**	**0.04**	**0.004**	R2 = 0.025 (SD 0.016)	**Vegetables**	**0.000**	**0.00 (0.00, 0.00)**	**0.03**	**0.033**
	**Processed meats**	**-0.023**	**-0.04 (-0.09, 0.00)**	**-2.99**	**0.038**		Vitamin B3	-0.012	-0.05 (-0.12, 0.01)	-3.53	0.112
	Vitamin B3	-0.011	-0.06 (-0.12, 0.00)	-3.98	0.058		FeH	-0.019	-0.03 (-0.08, 0.01)	-2.33	0.154
	Vitamin C	-0.009	-0.03 (-0.07, 0.01)	-2.08	0.124		Cholesterol	-0.044	-0.06 (-0.15, 0.03)	-4.17	0.165
	FeH	-0.019	-0.03 (-0.08, 0.01)	-2.18	0.178		Vitamin C	-0.009	-0.03 (-0.07, 0.01)	-1.74	0.218
	Vitamin B5	-0.006	-0.05 (-0.13, 0.03)	-3.44	0.226		Alpha-linolenic acid	0.008	0.03 (-0.02, 0.08)	2.14	0.218
	Red meat and offal	-0.016	-0.03 (-0.08, 0.02)	-2.02	0.247		Processed meats	-0.021	-0.03 (-0.07, 0.02)	-1.97	0.219
	Cholesterol	-0.041	-0.05 (-0.14, 0.04)	-3.41	0.250		Vitamin B1	0.000	-0.04 (-0.10, 0.03)	-2.61	0.248
	Animal protein	-0.017	0.04 (-0.04, 0.11)	2.55	0.324		Animal protein	-0.014	0.04 (-0.03, 0.11)	2.71	0.303
	Alpha-linolenic acid	0.008	0.02 (-0.03, 0.08)	1.71	0.355		Plant protein	0.011	0.04 (-0.04, 0.13)	3.13	0.325
	Legumes	0.008	0.02 (-0.02, 0.06)	1.34	0.361		Vitamin B5	-0.004	-0.04 (-0.12, 0.04)	-2.67	0.356
	Eggs	-0.001	-0.02 (-0.08, 0.03)	-1.57	0.411		Red meat and offal	-0.014	-0.02 (-0.07, 0.03)	-1.55	0.381
	Mn	0.002	0.02 (-0.03, 0.07)	1.42	0.444		Caffeine	-0.016	-0.02 (-0.07, 0.03)	-1.40	0.385
	Vitamin E	0.007	0.03 (-0.04, 0.09)	1.76	0.460		Beta-carotene	0.000	0.02 (-0.03, 0.07)	1.44	0.405
	Plant protein	0.008	0.03 (-0.05, 0.11)	2.06	0.493		Eggs	-0.003	-0.02 (-0.08, 0.03)	-1.48	0.446
	Vitamin B2	-0.009	-0.02 (-0.10, 0.05)	-1.50	0.567		Legumes	0.008	0.02 (-0.03, 0.06)	1.11	0.453
	Linoleic acid	0.002	0.02 (-0.05, 0.09)	1.30	0.588		Mn	0.001	0.02 (-0.04, 0.07)	1.15	0.538
	Caffeine	-0.009	0.00 (-0.05, 0.04)	-0.31	0.840		Vitamin E	0.002	0.02 (-0.05, 0.08)	1.11	0.638
	Plant lipids	0.007	-0.01 (-0.09, 0.07)	-0.49	0.863		Plant lipids	0.015	0.02 (-0.06, 0.09)	1.17	0.667
	Arachidonic acid	-0.022	0.00 (-0.06, 0.07)	0.24	0.913		Sweetened soft drinks	0.000	-0.01 (-0.05, 0.03)	-0.57	0.692
							Vitamin B2	-0.010	-0.01 (-0.09, 0.07)	-0.92	0.739
							Arachidonic acid	-0.018	0.01 (-0.05, 0.07)	0.63	0.782
**Principal Component 09**	(n=1)					**Principal Component 09**	(n=21)				
α=1, λ=0.031	**Oilseeds**	**-0.021**	**-0.07 (-0.10, -0.03)**	**-4.58**	**0.0003**	α=0.1, λ=0.111	**Docosapentaenoic acid**	**0.023**	**0.05 (0.01, 0.09)**	**3.64**	**0.009**
RMSE = 0.99 (SD 0.03)						RMSE = 0.989 (SD 0.025)	**Beta-carotene**	**0.026**	**0.06 (0.01, 0.10)**	**4.00**	**0.010**
R2 = 0.028 (SD 0.016)						R2 = 0.027 (SD 0.016)	Cu	-0.020	-0.04 (-0.08, 0.01)	-2.48	0.093
							Oilseeds	-0.035	-0.04 (-0.09, 0.01)	-2.67	0.098
							Processed meats	0.012	0.03 (-0.01, 0.08)	2.46	0.100
							Complex Carbs	0.012	0.06 (-0.01, 0.13)	4.13	0.110
							Poultry	0.007	0.03 (-0.01, 0.07)	2.23	0.117
							Red meat and offal	0.007	0.03 (-0.01, 0.08)	2.28	0.144
							Iodine	-0.009	-0.02 (-0.06, 0.01)	-1.68	0.190
							Vitamin B5	-0.005	-0.05 (-0.13, 0.03)	-3.33	0.239
							Vitamin K	0.007	0.02 (-0.02, 0.07)	1.64	0.283
							Salty snacks	0.005	0.02 (-0.02, 0.06)	1.46	0.290
							Breakfast cereals	-0.013	-0.02 (-0.06, 0.02)	-1.51	0.303
							Whole grains	-0.006	-0.02 (-0.06, 0.02)	-1.17	0.399
							Sugar and confectionary	-0.007	-0.02 (-0.06, 0.03)	-1.31	0.406
							Cakes and biscuits	0.004	0.02 (-0.03, 0.06)	1.33	0.417
							Ca	-0.006	-0.02 (-0.08, 0.05)	-1.28	0.579
							Vitamin B2	-0.004	0.02 (-0.06, 0.11)	1.47	0.628
							Plant lipids	-0.012	-0.02 (-0.09, 0.05)	-1.17	0.634
							Simple Carbs	-0.014	-0.02 (-0.08, 0.05)	-1.12	0.642
							Milk and soft cheeses	-0.003	-0.01 (-0.05, 0.04)	-0.68	0.666

Acronyms: CI, confidence interval, HR, hazards ratio; RMSE, root mean squared error.
ElasticNet results for identifying dietary features (nutrients, food groups) associated with each metabolite found to be associated with breast cancer. For each metabolite, selected dietary variables (nutrients/foods) were then jointly fit as predictors to estimate their direct associations, reported as β coefficients with 95% CIs.
To facilitate interpretation, the coefficient was exponentiated (2^coef) to reverse the log2 transformation applied to the metabolite levels. The coefficient 2^coef represents the direct change in metabolite levels for a one standard deviation increase in the dietary variable, while the raw coefficient corresponds to the fold change.
Model 1–S1 was adjusted for age at baseline (continuous), waist circumference (continuous), height (continuous), education level (< high-school degree, ≤ 3 years of higher education, >3 years of higher education), family history of cancer (all types, immediate family; yes/no), prevalent health condition at baseline or diagnosed within one year follow-up (includes type II diabetes, hypertension, hypercholesterolemia), smoking status (never, former, current) and smoking pack-years (continuous), alcohol intake (g/day), physical activity (IPAQ categories: low, medium, high), age at menarche (<12, 12–15, >15 years), number of biological children (continuous), age at first birth (no children, <20 year, 20–24, 25–34, ≥35 years), contraceptive use (ever; yes/no), menopausal status at baseline (pre/postmenopausal), number of R24H (continuous), time from last R24H to baseline (continuous), and energy intake (kcal/day excluding alcohol).
Model 1–S3 was model 1–S1 except excluding participants with extreme energy intake outliers (<928 or >2947 kcal/day, corresponding to the 1st and 99th percentiles).

Supplementary Table 6. Mediation analysis of significant diet–metabolite associations to estimate the total, direct and indirect effects of dietary variables on breast cancer risk with metabolites acting as mediators, NutriNet-Santé case-cohort study, N=2993, 2011-2022.

			Direct Effect Controlling for Mediator (RCDE)		Direct Effect Not Controlling for Mediator (RPNDE)		Total Direct Effect (RTNDE)		Indirect Effect by Mediator (RPNIE)		Total Indirect Effect (RTNIE)		Total Effect of Diet (RTE)		Proportion of Total Effect Mediated (PM)	
**Mediator**	**Dietary Feature**	**Model**		**p value**	**HR (95% CI)**	**p value**	**HR (95% CI)**	**p value**	**HR (95% CI)**	**p value**	**HR (95% CI)**	**p value**	**HR (95% CI)**	**p value**	**Proportion**	**p value**
**Glycohyocholic acid**																
	**Vitamin K**	**Main**	**0.825 (0.684–0.940)**	***0.010***	**0.825 (0.684–0.940)**	***0.010***	**0.825 (0.684–0.940)**	***0.010***	**0.991 (0.979–0.998)**	***0.014***	**0.991 (0.979–0.998)**	***0.014***	**0.817 (0.677–0.929)**	***0.006***	**0.042 (0.006–0.165)**	***0.020***
		Postmenopausal	0.812 (0.673–0.939)	***0.004***	0.812 (0.673–0.939)	***0.004***	0.812 (0.673–0.939)	***0.004***	0.994 (0.985–1.001)	0.114	0.994 (0.985–1.001)	0.114	0.807 (0.670–0.934)	***0.002***	0.024 (-0.004–0.107)	0.112
	Processed meats	Main	0.989 (0.854–1.135)	0.910	0.989 (0.854–1.135)	0.910	0.989 (0.854–1.135)	0.910	0.991 (0.980–0.999)	***0.020***	0.991 (0.980–0.999)	***0.020***	0.980 (0.844–1.126)	0.826	0.452 (-1.737–2.053)	0.826
		Postmenopausal	0.975 (0.827–1.155)	0.712	0.975 (0.827–1.155)	0.712	0.975 (0.827–1.155)	0.712	0.995 (0.984–1.003)	0.216	0.995 (0.984–1.003)	0.216	0.971 (0.821–1.150)	0.684	0.161 (-0.605–0.727)	0.716
	Plant lipids	Main	0.967 (0.805–1.133)	0.666	0.967 (0.805–1.133)	0.666	0.967 (0.805–1.133)	0.666	1.009 (1.000–1.024)	***0.040***	1.009 (1.000–1.024)	***0.040***	0.976 (0.816–1.144)	0.766	-0.374 (-1.533–2.009)	0.774
		Postmenopausal	1.010 (0.830–1.215)	0.888	1.011 (0.830–1.215)	0.888	1.011 (0.830–1.215)	0.888	1.009 (0.998–1.025)	0.120	1.009 (0.998–1.025)	0.120	1.020 (0.835–1.225)	0.816	0.443 (-1.017–1.789)	0.836
	Refined grains	Main	1.054 (0.908–1.223)	0.492	1.054 (0.908–1.223)	0.492	1.054 (0.908–1.223)	0.492	1.010 (1.002–1.025)	***0.014***	1.010 (1.002–1.025)	***0.014***	1.065 (0.915–1.233)	0.412	0.169 (-1.224–1.732)	0.410
		Postmenopausal	1.056 (0.902–1.241)	0.488	1.056 (0.902–1.241)	0.488	1.056 (0.902–1.241)	0.488	1.003 (0.995–1.014)	0.432	1.003 (0.995–1.014)	0.432	1.059 (0.907–1.245)	0.466	0.050 (-0.446–0.535)	0.670
**Linoleyl-carnitine**																
	Breakfast cereals	Main	1.016 (0.874–1.161)	0.804	1.016 (0.874–1.161)	0.804	1.016 (0.874–1.161)	0.804	0.989 (0.975–1.000)	***0.046***	0.989 (0.975–1.000)	***0.046***	1.005 (0.864–1.148)	0.900	-2.109 (-2.662–2.167)	0.906
	Milk and soft cheeses	Main	0.997 (0.878–1.124)	0.976	0.997 (0.878–1.124)	0.976	0.997 (0.878–1.124)	0.976	1.010 (1.001–1.021)	***0.036***	1.010 (1.001–1.021)	***0.036***	1.006 (0.886–1.137)	0.908	1.551 (-2.212–2.375)	0.912
	Potatoes and tubers	Main	0.988 (0.829–1.162)	0.850	0.988 (0.829–1.162)	0.850	0.988 (0.829–1.162)	0.850	0.997 (0.990–1.002)	0.312	0.997 (0.990–1.002)	0.312	0.985 (0.828–1.160)	0.832	0.176 (-0.496–0.480)	0.860
	Yogurt	Main	0.943 (0.791–1.110)	0.452	0.943 (0.791–1.110)	0.452	0.943 (0.791–1.110)	0.452	1.011 (1.000–1.026)	0.054	1.011 (1.000–1.026)	0.054	0.953 (0.804–1.124)	0.546	-0.222 (-1.815–2.027)	0.576
	Cakes and biscuits	Main	0.882 (0.733–1.022)	0.098	0.882 (0.733–1.022)	0.098	0.882 (0.733–1.022)	0.098	0.991 (0.979–1.000)	***0.042***	0.991 (0.979–1.000)	***0.042***	0.874 (0.724–1.013)	0.070	0.063 (-0.094–0.546)	0.104
	Vitamin E	Main	1.019 (0.844–1.220)	0.890	1.019 (0.844–1.220)	0.890	1.019 (0.844–1.220)	0.890	0.981 (0.958–0.998)	***0.034***	0.981 (0.958–0.998)	***0.034***	0.999 (0.823–1.195)	0.918	111.49 (-3.776–3.399)	0.924
**Glycoursodeoxycholic acid**																
	Oilseeds	Main	1.018 (0.881–1.156)	0.822	1.018 (0.881–1.156)	0.822	1.018 (0.881–1.156)	0.822	0.993 (0.982–1.001)	0.096	0.993 (0.982–1.001)	0.096	1.011 (0.875–1.146)	0.906	-0.683 (-2.002–1.992)	0.918
		Postmenopausal	0.984 (0.807–1.137)	0.844	0.984 (0.807–1.137)	0.844	0.984 (0.807–1.137)	0.844	0.990 (0.976–0.999)	***0.022***	0.990 (0.976–0.999)	***0.022***	0.974 (0.799–1.125)	0.750	0.385 (-2.548–1.903)	0.752
	Vitamin E	Main	0.992 (0.832–1.167)	0.890	0.9924 (0.832–1.167)	0.890	0.9924 (0.832–1.167)	0.890	1.005 (0.999–1.016)	0.180	1.005 (0.999–1.016)	0.180	0.997 (0.836–1.171)	0.942	-1.886 (-1.311–1.057)	0.950
		Postmenopausal	1.043 (0.850–1.257)	0.694	1.043 (0.850–1.257)	0.694	1.043 (0.850–1.257)	0.694	1.008 (1.000–1.027)	0.074	1.008 (1.000–1.027)	0.074	1.052 (0.861–1.267)	0.628	0.170 (-1.491–1.323)	0.634
	Decosapentaenoic acid	Main	1.125 (0.994–1.243)	0.058	1.125 (0.994–1.243)	0.058	1.125 (0.994–1.243)	0.058	1.001 (0.997–1.008)	0.572	1.001 (0.997–1.008)	0.572	1.126 (0.996–1.245)	0.054	0.012 (-0.056–0.125)	0.590
		Postmenopausal	1.135 (1.004–1.286)	0.036	1.135 (1.004–1.286)	0.036	1.135 (1.004–1.286)	0.036	1.001 (0.993–1.010)	0.790	1.001 (0.993–1.010)	0.790	1.136 (1.006–1.284)	0.038	0.007 (-0.103–0.174)	0.804
	Complex Carbs	Main	0.924 (0.772–1.091)	0.378	0.924 (0.772–1.091)	0.378	0.924 (0.772–1.091)	0.378	1.012 (0.999–1.028)	0.082	1.012 (0.999–1.028)	0.082	0.935 (0.782–1.108)	0.456	-0.166 (-1.811–1.746)	0.518
		Postmenopausal	0.957 (0.777–1.159)	0.696	0.957 (0.777–1.159)	0.696	0.957 (0.777–1.159)	0.696	1.015 (1.001–1.037)	***0.034***	1.015 (1.001–1.037)	***0.034***	0.971 (0.791–1.178)	0.818	-0.483 (-1.619–2.742)	0.828
	Docosahexaenoic acid	Main	1.106 (0.980–1.240)	0.102	1.106 (0.980–1.240)	0.102	1.106 (0.980–1.240)	0.102	0.996 (0.989–1.001)	0.166	0.996 (0.989–1.001)	0.166	1.102 (0.974–1.235)	0.116	-0.039 (-0.285–0.211)	0.266
		Postmenopausal	1.097 (0.972–1.236)	0.136	1.097 (0.972–1.236)	0.136	1.097 (0.972–1.236)	0.136	0.993 (0.982–1.001)	0.102	0.993 (0.982–1.001)	0.102	1.090 (0.966–1.231)	0.172	-0.078 (-0.758–0.638)	0.258
**Taurocholic acid**																
	Soups and broths	Main	0.990 (0.842–1.162)	0.880	0.990 (0.842–1.162)	0.880	0.990 (0.842–1.162)	0.880	1.007 (1.000–1.018)	***0.044***	1.007 (1.000–1.018)	***0.044***	0.997 (0.852–1.169)	0.970	-2.361 (-0.838–1.594)	0.978
	Breakfast cereals	Main	1.002 (0.856–1.140)	0.998	1.002 (0.856–1.140)	0.998	1.002 (0.856–1.140)	0.998	1.006 (1.000–1.018)	***0.046***	1.006 (1.000–1.018)	***0.046***	1.009 (0.861–1.148)	0.938	0.751 (-1.363–1.461)	0.936
**Chenodeoxycholic acid**																
	Beta-carotene	Excl. early diagnosis cases	0.925 (0.738–1.101)	0.410	0.925 (0.738–1.101)	0.410	0.925 (0.738–1.101)	0.410	0.989 (0.976–1.000)	***0.036***	0.989 (0.976–1.000)	***0.036***	0.916 (0.736–1.088)	0.336	0.115 (-1.160–1.184)	0.368
	Oilseeds	Excl. early diagnosis cases	0.984 (0.807–1.150)	0.842	0.984 (0.807–1.150)	0.842	0.984 (0.807–1.150)	0.842	1.010 (1.001–1.026)	***0.042***	1.010 (1.001–1.026)	***0.042***	0.994 (0.818–1.158)	0.932	-1.852 (-1.830–1.763)	0.934
	Milk and soft cheeses	Excl. early diagnosis cases	1.060 (0.909–1.240)	0.400	1.060 (0.909–1.240)	0.400	1.060 (0.909–1.240)	0.400	1.003 (0.998–1.012)	0.264	1.003 (0.998–1.012)	0.264	1.064 (0.914–1.246)	0.374	0.058 (-0.306–0.438)	0.510
	Cu	Excl. early diagnosis cases	0.948 (0.728–1.100)	0.554	0.948 (0.728–1.100)	0.554	0.948 (0.728–1.100)	0.554	1.006 (0.999–1.019)	0.086	1.006 (0.999–1.019)	0.086	0.955 (0.733–1.107)	0.616	-0.140 (-1.311–1.411)	0.658
**LysoPC(18:1)**																
	Linoleic acid	Premenopausal	0.754 (0.319–1.348)	0.366	0.754 (0.319–1.348)	0.366	0.754 (0.319–1.348)	0.366	1.010 (0.980–1.051)	0.476	1.010 (0.980–1.051)	0.476	0.761 (0.328–1.365)	0.380	-0.032 (-0.422–0.469)	0.720
	Potatoes and tubers	Premenopausal	0.841 (0.489–1.240)	0.382	0.841 (0.489–1.240)	0.382	0.841 (0.489–1.240)	0.382	0.987 (0.949–1.014)	0.328	0.987 (0.949–1.014)	0.328	0.831 (0.481–1.213)	0.348	0.063 (-0.787–0.820)	0.564
	Cakes and biscuits	Premenopausal	0.834 (0.459–1.319)	0.386	0.834 (0.459–1.319)	0.386	0.834 (0.459–1.319)	0.386	0.976 (0.928–1.003)	0.110	0.976 (0.928–1.003)	0.110	0.820 (0.447–1.281)	0.336	0.109 (-0.946–0.981)	0.402
	Caffeine	Premenopausal	1.330 (0.666–1.941)	0.458	1.330 (0.666–1.941)	0.458	1.330 (0.666–1.941)	0.458	0.992 (0.954–1.019)	0.480	0.992 (0.954–1.019)	0.480	1.320 (0.657–1.914)	0.460	-0.029 (-0.419–0.365)	0.680
**Principal Component 04**																
	Breakfast cereals	Main	0.996 (0.848–1.148)	0.894	0.996 (0.848–1.148)	0.894	0.996 (0.848–1.148)	0.894	1.009 (1.000–1.021)	***0.040***	1.009 (1.000–1.021)	***0.040***	1.005 (0.853–1.154)	0.974	1.733 (-3.082–1.567)	0.970
	**Vitamin K**	**Main**	**0.826 (0.702–0.947)**	***0.008***	**0.826 (0.702–0.947)**	***0.008***	**0.826 (0.702–0.947)**	***0.008***	**0.991 (0.979–1.000)**	***0.050***	**0.991 (0.979–1.000)**	***0.050***	**0.819 (0.695–0.943)**	***0.002***	**0.041 (0.001–0.185)**	***0.048***
	Vegetables	Main	0.999 (0.998–1.001)	0.536	0.999 (0.998–1.001)	0.536	0.999 (0.998–1.001)	0.536	1.000 (1.000–1.000)	0.922	1.000 (1.000–1.000)	0.922	0.999 (0.998–1.001)	0.552	-0.009 (-0.319–0.530)	0.994
	Processed meats	Main	0.991 (0.856–1.129)	0.850	0.991 (0.856–1.129)	0.850	0.991 (0.856–1.129)	0.850	0.992 (0.979–1.000)	***0.044***	0.992 (0.979–1.000)	***0.044***	0.983 (0.849–1.117)	0.760	0.496 (-1.887–1.896)	0.788
**Principal Component 09**																
	Oilseeds	Excl. early diagnosis cases	0.985 (0.793–1.167)	0.840	0.985 (0.793–1.167)	0.840	0.985 (0.793–1.167)	0.840	1.009 (1.001–1.023)	***0.018***	1.009 (1.001–1.023)	***0.018***	0.994 (0.801–1.175)	0.908	-1.608 (-1.265–1.220)	0.906

Decosapentaenoic acid	Excl. early diagnosis cases	1.174 (1.004–1.329)	0.04	1.174 (1.004–1.329)	0.044	1.174 (1.004–1.329)	0.044	0.993 (0.981–1.000)	***0.046***	0.993 (0.981–1.000)	***0.046***	1.165 (0.998–1.321)	0.052	-0.051 (-0.228–0.024)	0.094
Beta-carotene	Excl. early diagnosis cases	0.926 (0.726–1.098)	0.410	0.926 (0.726–1.098)	0.410	0.926 (0.726–1.098)	0.410	0.991 (0.979–1.000)	0.064	0.991 (0.979–1.000)	0.064	0.918 (0.724–1.088)	0.368	0.099 (-1.086–0.854)	0.416

Acronyms: CI, confidence interval; HR, hazard ratio.

Results from mediation analysis conducted using a regression-based approach was applied to estimate the total, direct, and indirect effects of diet on breast cancer risk through metabolites as mediators. Nonparametric bootstrapping with 200 resamples was used to assess statistical significance for exploratory models, where indirect effects with $p < 0.05$ was considered statistically significant.

MAIN model was adjusted for age at baseline (continuous), waist circumference (continuous), height (continuous), education level (< high-school degree, ≤ 3 years of higher education, >3 years of higher education), family history of cancer (all types, immediate family; yes/no), prevalent health condition at baseline or diagnosed within one year follow-up (includes type II diabetes, hypertension, hypercholesterolemia), smoking status (never, former, current) and smoking pack-years (continuous), alcohol intake (g/day), physical activity (IPAQ categories: low, medium, high), age at menarche (<12, 12–15, >15 years), number of biological children (continuous), age at first birth (no children, <20 year, 20–24, 25–34, ≥35 years), contraceptive use (ever; yes/no), menopausal status at baseline (pre/postmenopausal), number of R24H (continuous), time from last R24H to baseline (continuous), energy intake (kcal/day excluding alcohol), and dietary patterns derived by principal component analysis.

Excluding Early Diagnosis Cases was the main model excluding cases diagnosed within the first two years of follow-up (n=86).

Postmenopausal and Premenopausal models were adjusted as in main model except excluding menopause status and recategorizing age of first birth (no children, <30, ≥30 years) to account for smaller sample sizes. Postmenopausal model was additionally adjusted for age at menopause and menopause hormone therapy use.
